# Six decades of animal accelerometry: trends, applications, and future directions

**DOI:** 10.1186/s40462-026-00641-1

**Published:** 2026-04-02

**Authors:** Jessica L. Rudd, Martin A. Collins, David Righton, Holly L. White, Matthew J. Witt, Serena Wright, Lucy A. Hawkes

**Affiliations:** 1 Hatherly Laboratories, Faculty of Health and Life Sciences, University of Exeter, Prince of Wales Road, Exeter, EX4 4PS UK; 2 British Antarctic Survey, NERC, High Cross, Cambridge, CB3 0ET UK; 3 Centre for Environment, Fisheries and Aquaculture Science, Pakefield Road, Lowestoft, NR33 0HT UK; 4 School of Environmental Sciences, University of East Anglia, Norwich, NR4 7TJ UK

**Keywords:** Accelerometer, Biologging, Machine learning, Precision livestock farming, Wildlife, Behavioural classification, Energy expenditure, Welfare

## Abstract

The use of animal-borne devices to measure acceleration has yielded insights into animal locomotion, behaviour and energy expenditure. We present the first systematic review of accelerometry on animals, spanning 60 years, across ecology, biomechanics, agriculture, neurosciences, medical and veterinary sciences from over 400 species and 1520 studies. Most work to date is on terrestrial mammals, particularly domesticated species, while aerial and aquatic animals are relatively under-represented owing to device size and attachment constraints, and just one study has been conducted on amphibians. Despite this, accelerometry research has expanded globally across nearly 100 countries, across all groups of animals including birds, fish, invertebrates, mammals, and reptiles, and across wild and captive animals, including domesticated and non-domesticated species. A variety of software has been used, with open-source software and, more recently, machine learning tools being employed. On-board processing of accelerometry data, including real-time behavioural classification with deep learning to improve accuracy, can reduce power consumption and memory use several-fold, increasing deployment duration. Transfer of device types, sensors and analytical routines between fields in accelerometry offers considerable opportunities for advancing the field. For example, precision livestock farming uses real-time welfare monitoring, which could be adapted for aquaculture or even wild animal monitoring in areas with good data connectivity, while medical sector health monitoring could be used for wild animal disease tracking. The rapid growth in accelerometry requires urgent collaborative approaches to address big data challenges. This includes establishing standardised data repositories, developing open-electronics for low-cost device development and bespoke sensors, fostering capacity building and technical collaboration for device and algorithm advancements. This review also highlights geographic inequities in research accessibility and authorship, with less than 6% of accelerometry studies originating from authors from Africa, South America and Asia (excluding Japan, which contributed the second most studies globally) combined. The field must prioritise translational and inclusive research practices and cross-disciplinary collaboration to maximise scientific value and foster innovation while maximising animal welfare.

**Supplementary Information:**

The online version contains supplementary material available at 10.1186/s40462-026-00641-1.

## Introduction

**Fig. 1:**
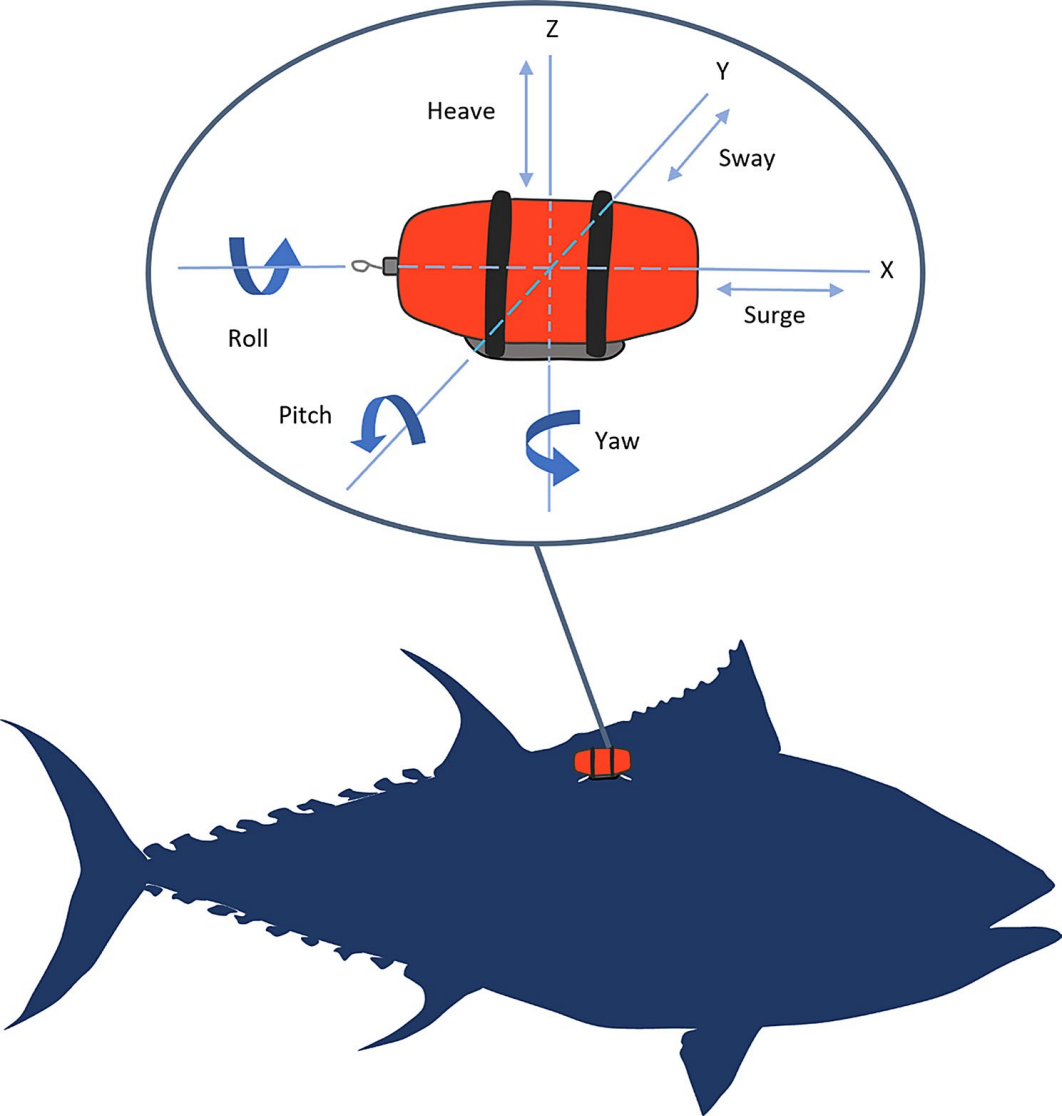
Graphical representation of a generic tri-axial accelerometer tag deployed on an Atlantic bluefin tuna (*Thunnus thynnus*), with inset of the tag’s three axes

**Fig. 2:**
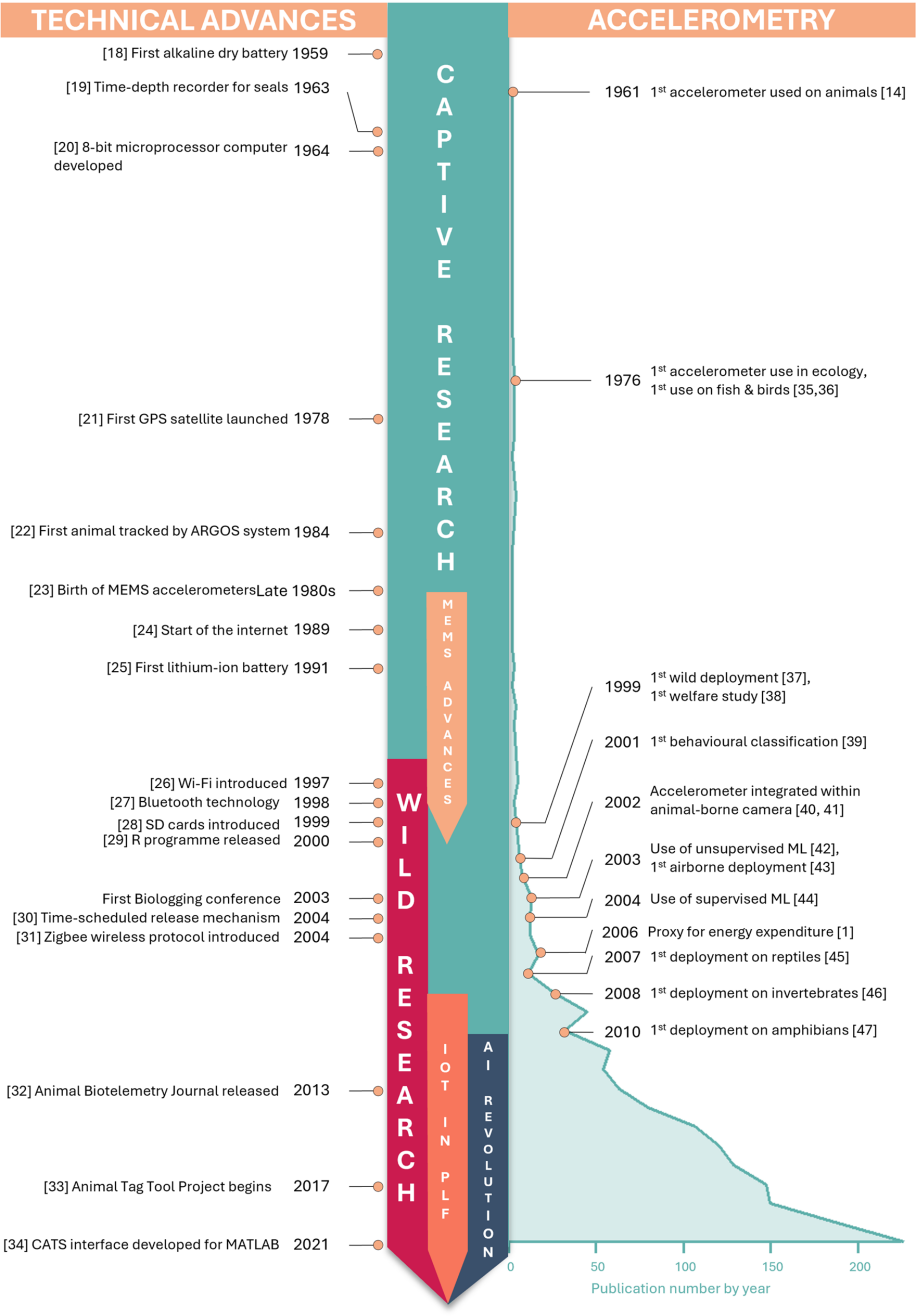
Summarised history of technological advances from the late 1950s to present day (left) and timeline of the number of accelerometry publications with key events in the field of animal accelerometry (right). IOT in PLF refers to advances in Internet of Things in precision livestock farming, MEMS refers to micro-electro mechanical systems. Figure references can be found in the reference list as entries: [1, 14, 18–47]

Movement is fundamental to life, and understanding how and why animals move is central to ecology and evolution, as well as a diverse range of research fields from agriculture to veterinary and biomedical sciences. Animal activity is defined largely in terms of movement, which results from muscular contractions requiring energy expenditure [1]. Since animal movement is typified by changes in speed in three dimensions, measurement of acceleration provides a direct quantification of activity, behaviour and the energy expended to produce it. Accelerometry, measured in m.s^−2^ or *g* (relating to units of gravity) across one to three axes (Fig. 1), comprises two components, (i) low-frequency “static” acceleration and (ii) high-frequency “dynamic” acceleration. The static component relates to the inclination of the accelerometer with respect to the earth’s gravitational field. It is typically measured using a smoothing window [2] or a lowpass filter [3] on raw acceleration data. Several factors can cause variation in the smoothed acceleration values beyond changes in static acceleration i.e. due to body pitch/roll. Smoothing the raw acceleration will produce values with a vector sum of 1 across three axes if the animal is moving at a constant velocity. In this scenario, changes in the smoothed acceleration values will reflect changes in body pitch/roll. There are three scenarios where the vector sum of the smoothed values can differ from 1 (which reflect other influences on on-board acceleration values): (i) the animal is free-falling (when the animal begins free-falling, the vector sum will be zero), (ii) the animal turns and experiences centripetal acceleration (e.g. [4]), (iii) the animal’s whole-body speed changes (i.e. it accelerates or decelerates in a linear fashion). Subtraction of the static data from the raw data in each corresponding axis leaves the dynamic acceleration, relating to the changes in velocity owing to the animal’s movement [5]. From these metrics, accelerometers enable behavioural classification [6], which can be quantified temporally into a time activity budget [7, 8]. Derived metrics (e.g. Vectorial Dynamic Body Acceleration (VeDBA) and Overall Dynamic Body Acceleration (ODBA)) correlate well with other proxies for energy expenditure such as respirometry, heart rate and doubly labelled water (DLW) [1, 5, 9–11], as muscular contraction to produce dynamic movement should dominate energy expenditure compared to resting metabolic rate and the thermic effect of food (but see [12]). These DBA-derived metrics offer advantages over traditional field metabolic rate measurements by providing longer-term continuous measurements without the temporal constraints of DLW, or invasiveness of heart rate loggers. In addition, DLW is not suitable for water-breathers such as fish owing to high rates of water turnover with their environment [1, 13]. The use of accelerometers in animal research began in the early 1960s to wirelessly monitor the behaviour of laboratory animals in less restricted, untethered settings [14], reducing stress and improving accuracy of classical behavioural conditioning studies, as well as in clinical research [15–17]. Since then, technological advancements including microprocessor miniaturisation, on-board memory storage and increased battery capacity have enabled the use of accelerometers in free-ranging animals, across a wide range of size classes and environments (Fig. 2). Early studies measured biomechanics, and transmitted activity remotely in captive mallards [35] and bluefish [36], which paved the way for the first wild study on free-ranging Adelie penguins [37]. Today, accelerometers are integral to biologging fields such as animal behaviour [48], energy expenditure [49], physiology [50], locomotion [51], social dynamics [52], response to environmental change [53] as well as anthropogenic disturbance [54], and there have been several reviews of the use of accelerometry over the last decade [9, 55–60].

More recently, accelerometers have become widely used in the agricultural sector, where efficient production of animals for food has become ever more important due to a growing global human population [61]. First introduced to monitor cow activity [62] and behaviour [63, 64], accelerometers are now a valuable component of precision livestock farming (PLF), integrating sensor technology and machine learning to improve animal productivity [65]. The PLF sector is projected to more than double from its 2019 value to reach a total market size of US$ 514 million by 2027 [66]. This has likely facilitated the commercial development of devices for PLF in disease detection [67] and behavioural classification [68, 69], including locomotion [70] recumbency [71, 72] and rumination [73]. The aquaculture sector is also undergoing rapid transformation as production of farmed seafood now surpasses global capture fisheries [74]. Accelerometers have been used to detect feeding in farmed fish [75] by incorporating artificial intelligence (AI) [76, 77], as well as investigating the effect of different stocking densities [78], delousing procedures [79], anthropogenic disturbances [80] and environmental conditions [81, 82].

Accelerometers are also being used to assess welfare, health, and physiology across a broad range of species in both captivity and the wild [81, 83–91]. Accelerometers are now commonly used to detect injuries [92], infection [93], behaviour following disease outbreaks [87, 94, 95], to monitor activity [96, 97], response to treatment [98–100], and detect events such as seizures [101] or parturition [102]. More recently, accelerometers have been used to understand the effect of tagging [103–106] and best practices for catch-and-release fisheries [107, 108].

Animals are still used as models to study human health [109], and accelerometry has fed into this discipline as well. For example, accelerometers can measure gait change in dogs with muscular dystrophy, which has helped evaluate disease progression and pre-clinical therapeutic trials for humans [110, 111]. Similarly, primate models of Parkinson’s disease have used accelerometers to monitor movement and treatment effects [112, 113]. Accelerometry has also been used to explore sport-related brain injuries using mice and rats [114], CPR quality during cardiac arrest simulations, and evaluating trauma responses to blast injuries using porcine models [115–117]. Accelerometers can also offer insights at the physiological level, including cardiac activity [118, 119], respiration rates to assess anaesthetic depth during surgery [120], and tremors and blood pressure changes [121], highlighting the significant role of accelerometry in medical and healthcare fields through animal models [122, 123].

To date there is no synthesis capturing the full cross-disciplinary breadth of applications of accelerometers in animal research. Much of the knowledge generated remains siloed within disciplines, limiting the transfer of technology, best practice and analytical techniques. Cross-disciplinary collaboration is particularly important as we continue to work in the era of ‘big data’ [124], and collaboration across engineers, biologists, computer scientists, ethicists and medical practitioners will maximise the utility of accelerometry. This review:

1. Provides a comprehensive, cross-disciplinary and taxonomic synthesis of accelerometry use across wild, captive, and domestic animal research.
2. Evaluates technological evolution and methodological landscape in accelerometry research, from traditional approaches to emerging AI and real-time processing capabilities.
3. Demonstrates cross-disciplinary knowledge transfer and collaborative approaches, highlighting how managed care research informs wild animal studies and vice versa.
4. Identifies geographic, taxonomic, and methodological gaps while proposing pathways toward more inclusive and accessible accelerometer research globally.

## Methods

### Query design and eligibility criteria

**Fig. 3:**
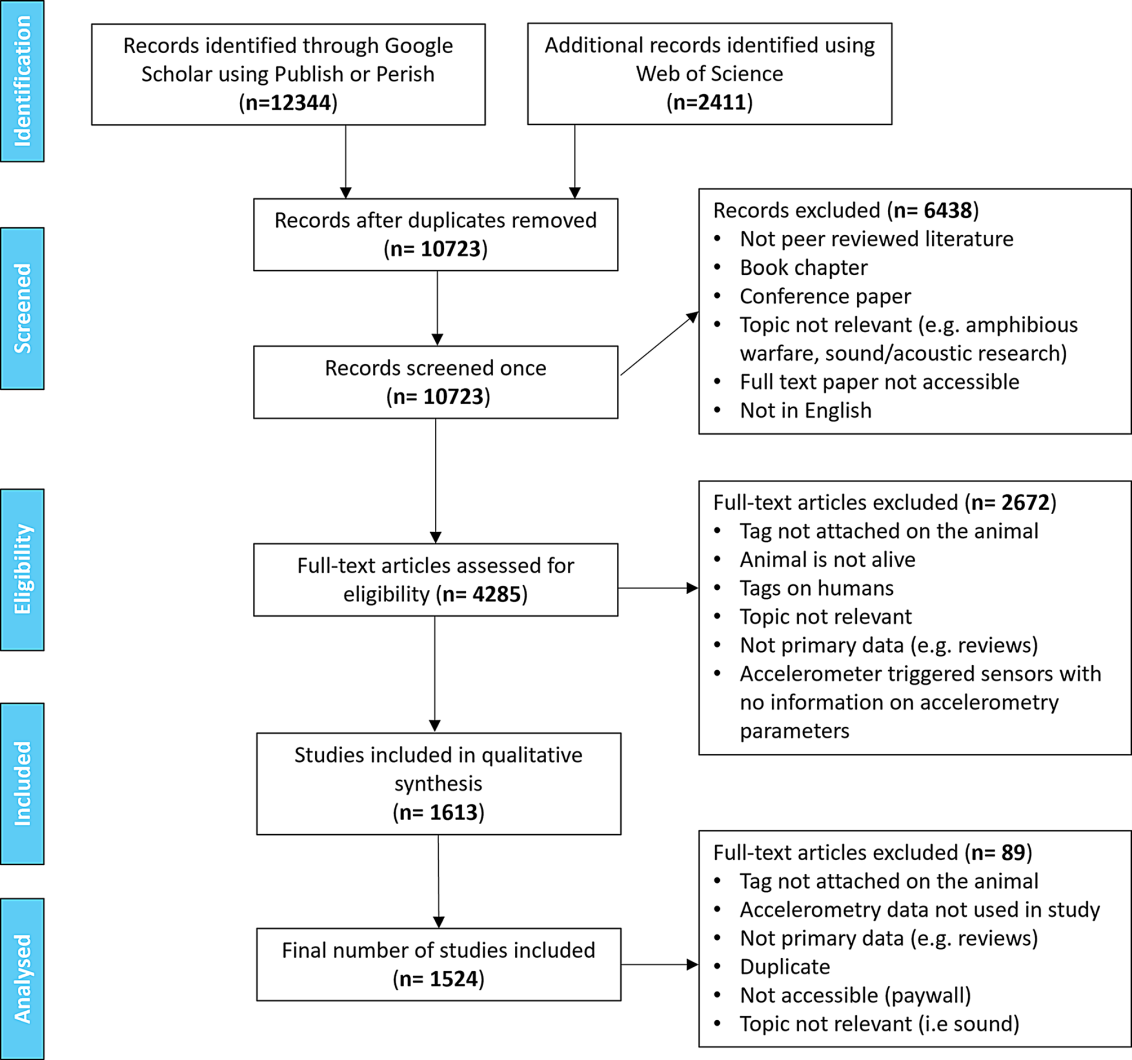
Preferred reporting items for systematic reviews and meta-analyses (PRISMA) flow diagram for the literature review on accelerometry use on live animals

All literature searches were conducted in February 2022, including records published between 1900 and 2021 (see Supplementary methods). Following the Preferred Reporting Items for Systematic Reviews and Meta-Analyses (PRISMA) guidelines [125], abstracts (*n* = 12,344) were filtered and resulting publications (*n* = 4,285) underwent full text assessment. Articles published between 1961 and 2021 proceeded to analysis (*n* = 1,524). Metrics (*n* = 32) were collected from these articles following standardised protocols (Fig. 3, Supplementary methods, Supplementary Tables 1 and 2). All publications and documented information were evaluated by a single reviewer (JR or HW).

### Analysis

#### Animal classification

For this review, animals were classified using two independent systems. First, species were divided into domestic (livestock, pets and model species, such as cows, pigs, horses, dogs, cats, mice, rats etc) and non-domestic (all wild species). Second, animals were further classified based on the study setting, as wild (free-ranging species in their natural environment) or captive (animals studied in enclosed settings such as zoos, aquaria, fenced enclosures or laboratories). These categories are independent, meaning non-domestic animals can be studied in either wild or captive settings, and domestic animals can similarly be found in both contexts. All references to mammalian research do not include human research.

#### Tag specificity

For each study, the use of additional sensors alongside accelerometers was recorded, with “sensor complexity” defined as the number of different sensor types used. To focus on the most commonly adopted technologies, only sensor types used in five or more publications were included in subsequent analyses. Changes in sensor complexity over time were examined separately for domestic and non-domesticated species, and between wild and captive animals.

#### Machine learning

To investigate trends in the application of Machine Learning (ML) in animal accelerometry studies, publications were grouped by ML approach, taxon, status (wild, captive and/or domesticated or non-domesticated) and broad research topic. The seven most used software programmes specifically used for ML analyses were recorded, and only algorithms (e.g. Random Forest, Support Vector Machine etc.) used in more than four publications were considered. This threshold was set to focus the analysis on the most widely adopted algorithms, as many were used only once across the reviewed literature and thus provided limited insight into broader trends. Algorithms were subsequently grouped into ten categories based on their fundamental mathematical frameworks and underlying computational approaches (e.g. Random Forest models and Classification and Regression Trees grouped under the “Decision Tree” category, Supplementary Table 3), allowing for comparison of classification models by broad research area. Trends in the use of supervised versus unsupervised ML were visualised using a Sankey diagram created with the R packages “networkD3” and “webshot”. A heat map created using the “geom_tile” function in ggplot was used to investigate the prevalence of each ML approach across research topic.

#### Global patterns of accelerometry use

Global patterns of use of accelerometry were visualised by first author country, by all listed authors’ affiliation countries, and by country of data collection, using the R packages “rnaturalearthdata”, “rnaturalearth” and “ggplot2”. The total number of publications was also grouped by world region, where data have been predominantly collected, and where listed authors were located. A chord diagram was produced using the R packages “chorddiag” and “circlize” to visualise publications in relation to all authors stated affiliations with the country where the data were collected (i.e. parachute science, defined as where research is conducted in a country without local authorship [126]).

## Results and discussion

**Fig. 4:**
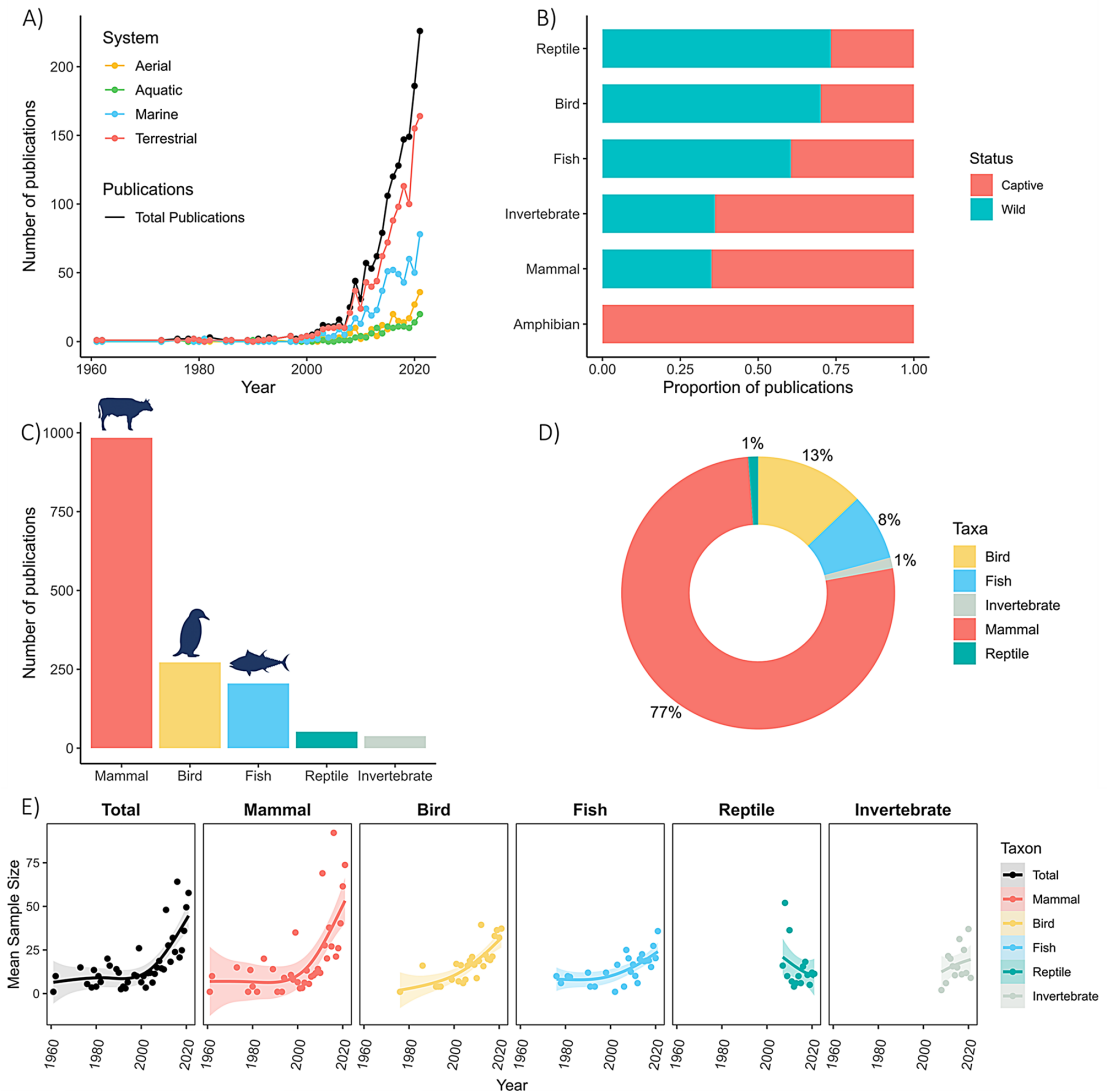
Taxonomic coverage of accelerometry research. (**A**) Time series showing the total number of publications per year (black line) along with the number of publications by geographic system: terrestrial (red), aquatic (green), marine (blue) and aerial (yellow). (**B**) Proportion of studies conducted on wild versus captive animals, separated by taxon. (**C**) Total number of publications by taxon. (**D**) Proportion of all tags deployed across publications, grouped by taxon. (**E**) Change in mean sample size over time for all studies (black), mammals (red), birds (yellow), fish (blue), reptiles (green) and invertebrates (grey)

The first study using accelerometry in animal research was published in 1961, while the number of publications per year rapidly increased from 2007, with over 100 articles published per year since 2015 and reaching 226 per year by the end of 2021 (Fig. 4A).

### Accelerometry research landscape

#### Taxonomic coverage and research focus

More than 54,200 accelerometers were deployed on animals throughout the study period, covering 477 species (Supplementary Tables 1 and 4). Nearly half of all species (48%, *n* = 228) appeared in just a single study, while only 12% of publications included more than two species in their study. Mammals were the most studied taxonomic group (189 species), followed by birds (126 species), fish (113 species), invertebrates (25 species), reptiles (23 species), and a single amphibian species. The domesticated cow was the most frequently studied, accounting for 38% of all deployed tags (*n* = 20,353) and 15% of all publications (*n* = 235). This was followed by dogs (*n* = 86 publications), sheep (*n* = 58), domestic horses (*n* = 54) and pigs (*n* = 49), with a total 36% (*n* = 565) of all studies conducted on domestic animals. For wild animals, Southern elephant seals (*n* = 29), humpback whales (*n* = 19), blue whales (*n* = 17), Northern elephant seals (*n* = 16) and green turtles (*n* = 14) were the most frequently studied species. Although fewer than half of all studies focused on wild animals (*n* = 740 studies, Fig. 4B), the proportion of wild to captive studies varied between taxonomic class (Fig. 4B). Captive studies dominated the literature for mammals (65%, *n* = 600 studies) and invertebrates (64%, *n* = 23, and the single amphibian study), whereas studies on wild animals were greater in fish (60%, *n* = 124), birds (72%, *n* = 177) and reptiles (73%, *n* = 33) (Fig. 4B). When excluding studies on domesticated mammals (e.g. cows, sheep, pigs, dogs and cats), the number of studies on wild mammals was nearly three times greater than captive mammals (wild studies *n* = 362, captive studies *n* = 132).

The number of animals tagged per study varied, with an average sample size of 36 animals ± 152 s.d. per study (range: 1–3649, see Supplementary Tables 1), varying by taxa (mean sample size mammals: 43 ± 6; birds: 26 ± 2; fish: 22 ± 3; invertebrates: 18 ± 3, reptiles: 15 ± 3; amphibians: 9) and by study topic (Supplementary Table 5). The average sample size increased over time by almost six-fold between the start of the review period and 2021 (mean: 58 ± 14), a trend followed by all taxa to a varying degrees, except for reptiles, which decreased over time (Fig. 4E). Mammals accounted for the largest share of accelerometers deployed across all studies combined (77%, Fig. 4D). Terrestrial studies dominated the literature (Fig. 4A), comprising 72% of all publications in 2021 for example. While studies on marine, freshwater and aerial species have increased over time, they continue to lag, accounting for 35, 9 and 16% of publications in 2021 respectively. This may reflect the unique logistical challenges associated with studying animals in these environments. Factors such as drag, buoyancy and tag load [104, 127–130] can affect device miniaturisation owing to technical issues including waterproofing, pressure resistance and tag recovery. 

Device size and attachment remain a key constraint [131], particularly for smaller or sensitive species such as those with exposed skin or other soft structure [132–134]. As a result, there is a lack of research on non-commercially important invertebrate species [46, 50, 135, 136]. Only one study has been published on amphibians, focusing on cane toads (*Bufo marinus*) [47], though a second study on European green toad (*Bufotes viridis*) has been published since the review period [137]. This knowledge gap is particularly concerning given that many of these understudied groups are amongst the most vulnerable to environmental change, making accelerometry-based insights into their behaviour and physiology increasingly critical for effective conservation management.

### Research topics and field specific trends

**Fig. 5:**
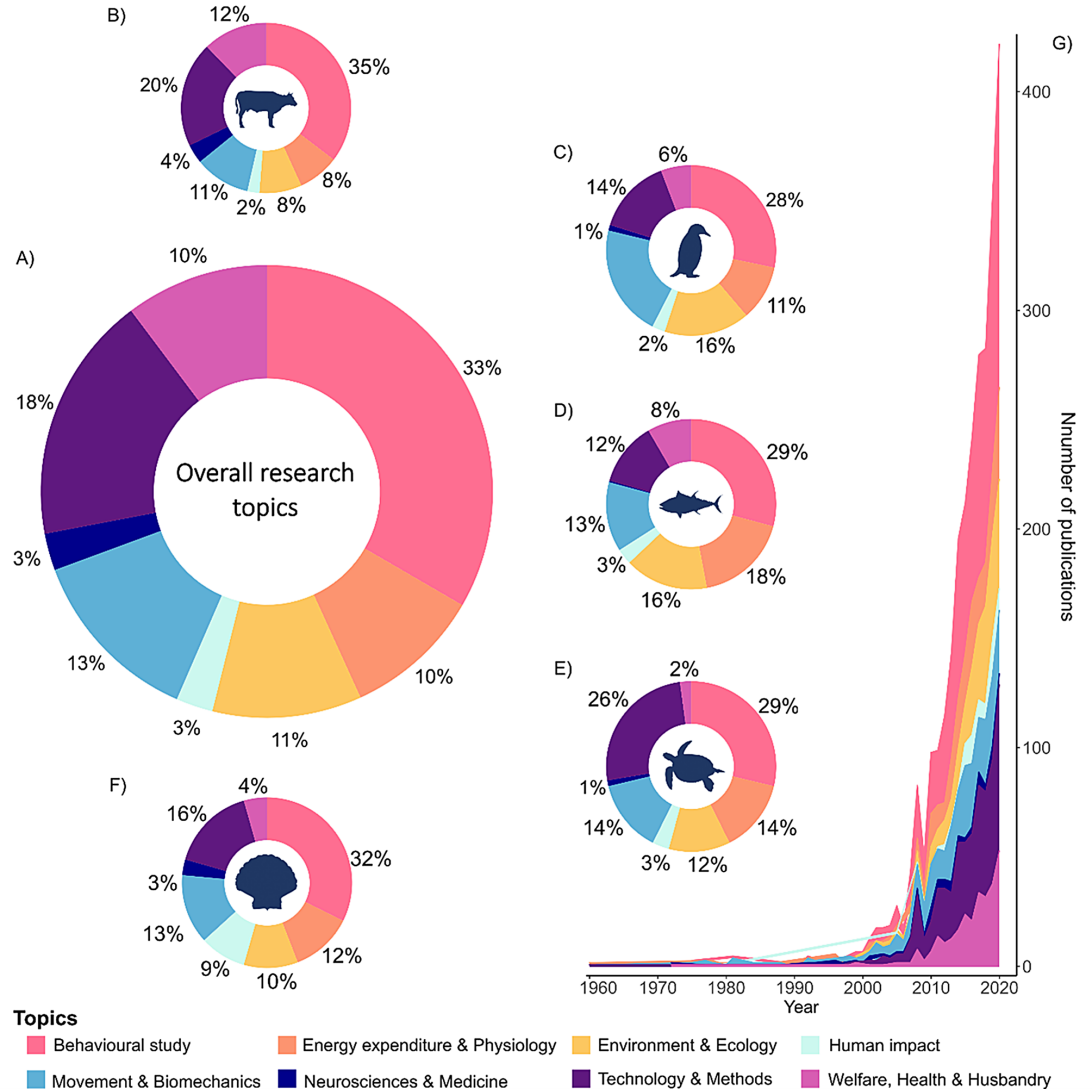
Trends in research topics. (**A**) Ring plot of the overall proportion of publications by research topic, further separated by animal class, with mammals in (**B**), birds (**C**), fish (**D**), reptiles (**E**) and invertebrates (**F**). (**G**) Stacked time series of the number of publications by research topic by year

Accelerometry research spanned over 100 topics across multiple disciplines, which we grouped into eight categories (Supplementary Table 2). Behavioural studies comprised a third of publications (*n* = 937) and was the most researched area for all taxa, ranging between 28% of publications for birds and 35% for mammals (Fig. 5). Studies focused on technology and methods (e.g. sensor development, tag attachment comparison, algorithm performance) were the next most common (18%), followed by movement and biomechanics (13%), environmental and ecological studies (11%), energy expenditure and physiology (10%), welfare, health & husbandry (10%), human impact (3%) and neuroscience and medicine (3%) (Fig. 5A). Studies on fish commonly addressed energy expenditure and physiology (18%), as well as environmental and ecological themes (16%), while publications on birds focused on movement and biomechanics (21%), and environmental and ecological studies (16%, Fig. 5C–D). Mammalian research focused on technology and methods (20%) and welfare, health and husbandry (12%) (Fig. 5B). Reptile and invertebrate studies were concentrated on technology and methods (26 and 16% respectively), and movement and locomotion (14 and 13% respectively) (Fig. 5E–F).

Behavioural research was equally prevalent among wild (34%) and captive studies (33%), but studies on wild animals had greater emphasis on environmental and ecological applications (17%), movement and biomechanics (13%), and technology and methods (13%), whereas studies on captive animals focused more heavily on technology and methods (23%), welfare, health and husbandry (15%), and energy expenditure and physiology (9%), likely driven by demands in agriculture and aquaculture industries, and the growing uptake of digitised farming. Captive studies have also been used to validate and calibrate accelerometry metrics (e.g. VeDBA, ODBA) with other metrics like VO_2_ max, doubly labelled water and heart rate [49, 138–143].

Studies focusing on welfare, health and husbandry were limited in reptiles (2%), invertebrates (4%), birds (6%) and fish (8%) (Fig. 5). This may reflect poorer consideration of welfare for non-mammalian species. The first mammalian welfare accelerometry study was published in 1999 [38], the first in birds not until 2007 [94], fish in 2012 [144], invertebrates in 2016 [80] and reptiles in 2017 [145]. While the use of accelerometers to monitor welfare in captivity is gaining momentum (Fig. 5G; 15% of all captive studies), uptake in wild animal studies remains limited (3% of studies). In wild animals, studies include investigating tag effect [104, 146–148], post-release behaviour [105, 149–151], mortality [152–155], entanglement [145, 156], and health [157, 158] but it remains challenging to disentangle affected and “normal” unaffected behaviour. Conversely, domestic and captive animals may be more readily habituated to handling and wearing sensors, making studies more feasible.

### Technological landscapes and methodological approaches

#### Sensor technology and deployment

**Fig. 6:**
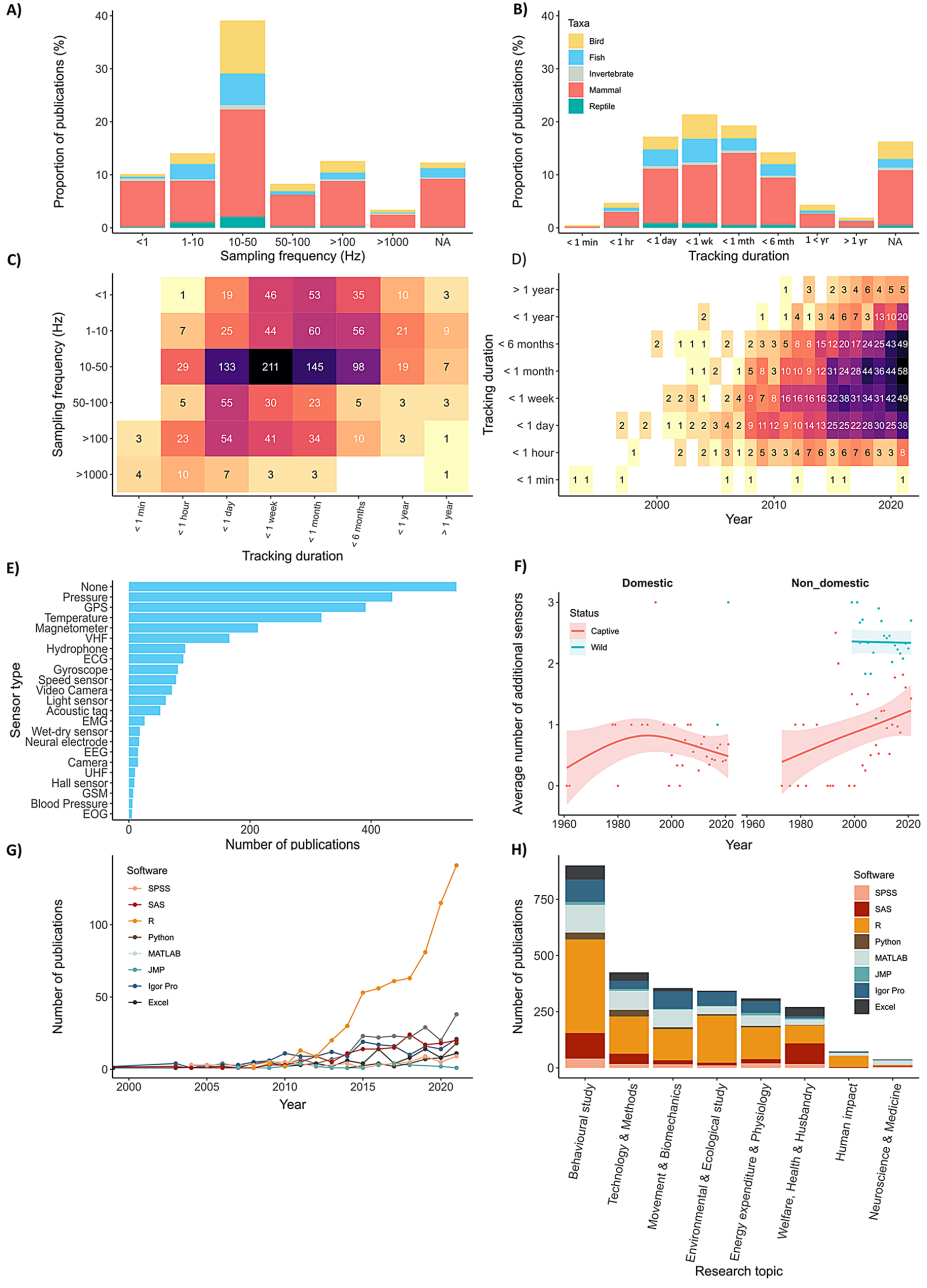
Technological landscape of accelerometry research. (**A**) Proportion of publications by sampling frequency range and (**B**) proportion of publications by tracking duration, coloured by animal class. (**C**) Heatmap of the number of publications by tracking duration and sampling frequency. (**D**) Heatmap of the number of publications by year and by tracking duration (only two publications prior to 1993 reported tracking durations, <1 month in 1976 and <1 hour in 1979). (**E**) Horizontal bar plot of the most common sensors used alongside accelerometers by number of publications. (**F**) Trend of the mean number of additional sensors used alongside accelerometers by year of publication for domestic (left) and non-domestic species (right), with distinction between captive (pink) and wild (blue) animals. Filled line represents ± the standard error. (**G**) Trends in software use over time, with number of publications by software use by year. (**H**) Trends in software use by research topic, illustrated by a stacked bar plot of the number of publications by research topic and by software

Over three quarters of all studies (76%) measured acceleration on 3-axes, 8% used 2-axes and 7% used only 1-axis (a further 8% did not specify the number of axes). For all taxa, the most common sampling frequency was 10–50 Hz (Fig. 6A; making up 39% of all publications). The second most common sampling frequency was < 1 Hz for invertebrates and mammals, 1–10 Hz for fish and reptiles, and >100 Hz for birds. Tracking duration ranged between < 1 min and 6 years [159], with the average (modal) deployment between 1 and 7 days (Fig. 6B). There was a general trend of decreasing deployment duration with increasing sampling frequency (Fig. 6C), though this relationship was not linear. The longest deployment durations used sampling frequencies of 1–10 Hz, likely reflecting a combination of factors including duty-cycled sampling (where high-frequency data collection occurs intermittently to extend battery life), variation in device size and battery capacity across different tag types, and research-specific deployment strategies where tracking duration was determined by the study objectives rather than device limitations. Tracking duration increased over time (Fig. 6D), with studies exceeding six months appearing after 2004, and those surpassing a year after 2011. Among the 33 publications with tracking duration longer than a year, extended duration was facilitated by either low sampling frequency [160–162], intermittent sampling at higher resolution [155, 163–166], transmission of summary statistics [167], or data collected over fewer axes [168–170].

While technological advances have enabled deployment durations exceeding a year, long-term device attachment raises ethical considerations. Devices can affect behaviour, health, and fitness over long temporal scales and be shaped by environmental conditions [171, 172]. In some cases, tag attachment can cause chronic physical damage including tissue damage and infection at attachment sites [171, 173], and behavioural and fitness consequences that may affect survival, reproduction and foraging success [174–176]. These require careful consideration, rigorous testing and transparent reporting of device effects with appropriate statistical power [177].

Two-thirds of all publications included at least one additional sensor. Pressure (indicating depth or altitude) was the most common (28% of studies), followed by GPS (26%), temperature (21%), magnetometry (14%), VHF (11%) and sound (hydrophone/microphone) (6%) (Fig. 6E). Wild animals were twice as likely to be instrumented with additional sensors compared to captive animals, and five times more likely than domestic species. Overall, sensor complexity has increased over time (linear regression: R^2^ = 0.0114, *p* < 0.001) with an average of 2 ± 1.98 sensors being deployed per publication by 2021. Tags with significantly more sensors were deployed on wild compared to captive individuals (Wilcoxon rank sum test W = 201440, *p* < 0.001 Fig. 6F), with an average 2.3 ± 1.7 additional sensors for wild animals (max = 9, on multiple whale species) and 0.79 ± 1.12 for captive animals (max = 6, on cows). While the average number of sensors per tag deployed on wild animals remained similar over time, there were marked differences between domesticated and non-domestic captive species, with simplification of device deployments in domesticated animals since the early 2000s, compared to a rapid increase in non-domestic captive animals (wild animals studied in captive settings such as laboratories, enclosures and aquaria) (Fig. 6F). This follows the development of off-the shelf tags (manufacturers such as Onset Computer Corporation and IceRobotics) in precision livestock farming, which generally only include an accelerometer.

While tag complexity has increased over time, particularly in wild animal studies, the welfare implications of this trend are difficult to assess. Device mass and size were inconsistently reported and depends on complex interactions between sensors, battery capacity, and design. For example, tags with identical sensors can vary 10-fold in mass based on battery [178], while adding a tri-axial accelerometer to a simple temperature-depth recorder can increase mass sixfold (2.7 *g* to 16.7 *g* in air [179, 180]). Welfare implications are multi-faceted and extend beyond simple mass ratios, as body mass is dynamic and may vary seasonally or depending on life history events, changing relative burden over the deployment duration [181]. Animals may also compensate for tag weight through body mass loss [182], undermining initial assessments based on initial deployment ratios. Actual forces exerted during movement can reach up to 54% of body mass in athletic species such as hunting cheetahs [104], while tag shape, attachment method and position can significantly influence behaviour, physiology and fitness independent of mass [171, 175, 176, 183, 184]. Miniaturisation has primarily enabled deployment of devices on smaller animals, rather than reducing relative burden [131], consistent with an increase in sensor complexity over time (Fig. 6F). These complexities highlight the need for better reporting of the of devices beyond simple mass ratios, as well as improved reporting of device specifications to enable future meta-analyses.

A substantial number of publications failed to specify methodological details such as sample size (*n* = 14), sampling frequency (*n* = 209) and tracking duration (*n* = 279), particularly in laboratory-based studies. There was also a lack of standardisation of terminologies related to activity metrics derived from acceleration data. While some metrics such as ODBA and VeDBA are clearly defined and consistently reported, other commonly used measures lack such uniformity. For instance, the terms *magnitude of acceleration*, *vector norm acceleration*, (*signal*) *vector magnitude*, *Euclidean norm* and *total acceleration* all describe the same underlying calculation, $\sqrt {a_x^2 + a_y^2 + a_z^2}$, but vary depending on the research field, device manufacturer or country of origin of the study. In addition, studies using devices such as those from ActiGraph, Analog Devices and Philips Respironics report only generic terms like “activity count” often without clarifying how the metric was derived or processed on-board, further complicating reproducibility and comparability across studies.

### Data processing and analysis methods

#### Software for analysis

Among the eight most commonly used programmes for data analysis, only R and Python were free and open-source. R was used in nearly half (42%) of all publications (*n* = 649), almost three times more than the second most used programme, MATLAB (*n* = 229, Fig. 6G). Whilst its first recorded use for accelerometry analysis was in 2008, R has dominated the field since 2011 (Fig. 6G), appearing in 62% of all papers published in 2021. This widespread adoption mirrors broader trends across biological sciences with Lai et al. [185] finding that over 60% of 60,000 ecology and evolutionary biology articles had used R, while Joo et al. [57] reported similar prevalence (65%) in movement ecology studies in 2018. The dominance of R is likely driven by its open accessibility and flexibility across a range of analytical needs, from data management and statistical modelling to data visualisation and machine learning. The growth in R’s use has occurred in parallel with substantial progress in the number and sophistication of quantitative methods for the study of movement. For instance, Joo et al. [186] identified 58 packages created to deal with tracking data, including accelerometry data, while Zhang et al. [187] reviewed R packages specifically designed to generate activity-related variables from accelerometry data. Several specialised packages have also been developed to extract and process accelerometry data from multiple tag manufacturers. The Animal Tag Tool Project (first launched in 2017; animaltags GitHub) was also developed to work across R, Python and MATLAB, enabling consistent data extraction protocols across platforms. Additionally, MATLAB has also been used to develop tools to integrate accelerometry with video output [34].

The prominence of R becomes more apparent when use is assessed by research area, being the choice for most environmental and ecological studies (70%), as well as studies focused on human impact (69%), energy expenditure and physiology (51%), behaviour (44%), and movement and biomechanics (38%) (Fig. 6H). In contrast, studies on domestic animals, which often use off-the-shelf tags, more commonly relied on proprietary software provided by the tag manufacturer (e.g. Hoboware Pro, IceTag Analyser, Rumiwatch Software). These typically include in-built behavioural classification algorithms and activity metrics, reducing the need for custom coding. For instance, among studies on cows that reported their choice of software, 44% relied on SAS compared to 27% that used R and 8% using MATLAB. Finally, MATLAB was most commonly used in medical and neuroscience research where compatibility with frequently used physiological sensors such as ECG and neural electrodes make it a more suitable platform. This reflects how software choice is often shaped not only by analytical preferences but also demands of the research context and the types of devices being integrated.

#### Machine learning

**Fig. 7:**
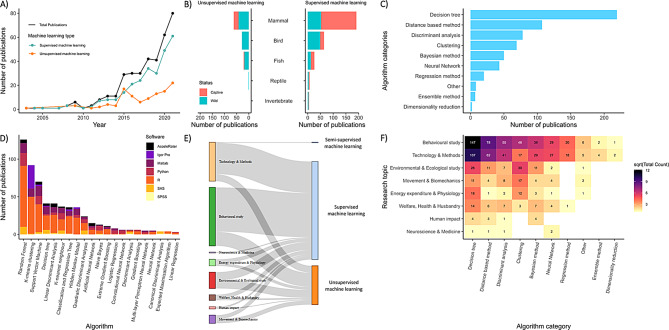
Machine learning applications in animal accelerometry data. (**A**) Time series of the total number of publications using ML (black), with distinctions between supervised (blue) and unsupervised (orange) approaches. (**B**) Stacked bar plot showing the number of publications by animal class, captivity status (captive vs wild) and type of ML approach. (**C**) Bar plot showing the number of publications by broad algorithm category. (**D**) Stacked bar plot of the number of publications by the most commonly used ML algorithm (used in > 4 publications) coloured by the seven most common software programmes for analysis. (**E**) Sankey diagram illustrating the relationship between learning approaches and broad research topics areas. The width of the lines corresponds to the number of publications linking each research topic to a given machine learning approach. (**F**) Heat map showing trends in broad algorithm use across research topic. Darker shades indicate a higher number of publications using a given algorithm within a topic area

Accelerometry data have been used to classify behaviour since its early use in wild animals [37], but it was not until 2003 that machine learning (ML) was initially used. ML can be defined as a computational approach that automatically learns patterns from data, so that it can make predictions or classifications on new, unseen, data without explicit programming. In a study on wild Weddell seals, unsupervised machine learning, which discovers hidden patterns in data without pre-labelled inputs by iteratively grouping similar observations together, was used on 58 spatio-temporal variables to classify diving behaviour [42]. Supervised approaches, which involves training a model using validated data where each input comes with a correct labelled output (for example, acceleration pattern X corresponds to behaviour A, while acceleration pattern Y corresponds to behaviour B), have been used since 2004 [44]. The algorithm learns to map inputs to outputs by finding a pattern in the labelled training data, then applies these learned relationships to make predictions on new, unlabelled data. Between 2003 and 2021, the use of ML to classify accelerometry data has increased (Fig. 7A), representing 361 publications across 182 species. This rapid growth has since prompted several comprehensive reviews of the field [188–192]. Supervised learning was the most commonly applied approach (74%, *n* = 268 studies), followed by unsupervised (30%, *n* = 107) and semi-supervised methods (*n* = 2). ML has been applied across all animal classes (Fig. 7B), the majority in mammals (64%, *n* = 230 publications), followed by birds (22%, *n* = 80), fish (11%, *n* = 41), reptiles (2%, *n* = 8) and invertebrates (1%, *n* = 4) where only supervised learning was used. ML has been more commonly used in wild than captive animals (*n* = 200 vs *n* = 188, Fig. 7B). However, 39% of all ML publications were on domestic species (*n* = 141), including the majority (*n* = 152 studies) of mammal studies.

Over 50 different ML algorithms were identified across the literature, broadly grouped into 10 categories (Supplementary Table 3), with decision trees, distance-based methods and discriminant analyses being the most common (Fig. 7C). On average, each study used more than one (mean 1.7 ± 1.4 s.d., max 10) algorithm to classify accelerometry data (1 ± 0.3 algorithm per publication for unsupervised approaches vs 1.8 ± 1.5 per publication for supervised approaches). Only 33% of these studies compared model performance, and 11% did not report model performance metrics. Validation remains one of the major challenges in using ML with accelerometry data [193, 194], particularly for rare or complex behaviours [195], coping with individual variability [196] and in field settings [197]. To address this, studies have incorporated additional sensors to help with the validation process [48, 198].

Publications using ML most commonly distinguished between general behaviours [199–201], including gait classification [202–204], swimming [134, 205] and flight [206–209], as well as foraging [210–212], reproduction [213–215], grooming [7], rest [216] and vigilance [217] (Fig. 7E). Technology and method-based studies followed next, reflecting algorithm development including on-board device classification [199, 218–221], model performance optimisation [222, 223] and surrogate species use [224, 225]. ML has also enabled links to be made between behaviour and environmental conditions [8, 226–228], seasonal patterns [229, 230], and animal-driven ecosystem impacts such as N_2_O emissions from livestock [231]. Applications also extended to welfare [201, 232, 233], health [93, 234] and husbandry [235, 236], as well as anthropogenic impacts [237] such as sound [238, 239], vessel disturbance [54, 103] and poaching [240]. Studies with a focus on energy expenditure and physiology used ML to develop time energy budgets [6, 241], investigate energy landscapes [242, 243], and measure the cost of parasitism [244]. In neuroscience and medical research, ML has helped to classify sleep-wake states and shows potential for detecting epileptic seizures and movement disorders such as Parkinson’s disease [101, 245, 246].

### Advanced analytics and artificial intelligence

The rapid advance of ML presents increasingly powerful and transformative opportunities for accelerometry. Deep learning, a subset of ML using multi-layered neural networks to automatically identify temporal patterns and extract hierarchical features in complex datasets, has emerged as a particularly promising new tool for analysing accelerometry data where traditional feature extraction methods may miss subtle behavioural signatures [191, 247, 248]. While deep learning has revolutionised data analysis across disciplines, this review (ending in 2021) captured only the beginning of its application to accelerometry, with 19 studies identified, 13 on captive animals, five on wild animals, and one on both, including six mammal species, four fish and two reptile species. These studies demonstrated that deep learning outperformed traditional ML algorithms in behavioural classification accuracy [199, 249–251]. In agricultural studies, deep learning facilitated real-time detection of behaviours such as drinking, feeding and oestrus in livestock [192, 249, 252], and enabled intelligent fish feeding systems in aquaculture [253]. It also showed great promise for analysing complex behaviours in free-ranging wildlife [250, 254, 255], such as identifying nesting behaviour in Mediterranean tortoises (*Testudo hermanni, Testudo graeca,* and *Testudo marginata*) [256] and classifying foraging behaviour in Canada lynx (*Lynx canadensis*) [255], narwhals (*Monodon monoceros*) [250] and green turtles (*Chelonia mydas*) where gyroscopes were more informative than accelerometers [254]. As deep learning continues to evolve, significant advances are expected in three key areas: (i) scalability across larger and more diverse datasets, (ii) cross-species generalisation, and (iii) enhanced real-time performance enabling data processing on-board devices [248]. These developments will likely enable researchers to quantify animal behaviour with unprecedented accuracy, detail and efficiency.

Although Large Language Models (LLMs) had not yet made their mark on accelerometry research by the end of our review period [257–260], they clearly have great potential to enhance interpretation of complex behavioural patterns in research from agriculture to disease monitoring, and wildlife response to changing environments, areas where rapidly growing volumes of sensor data and literature present both technical opportunities and analytical challenges.

### Geographic distribution and research accessibility

**Fig. 8:**
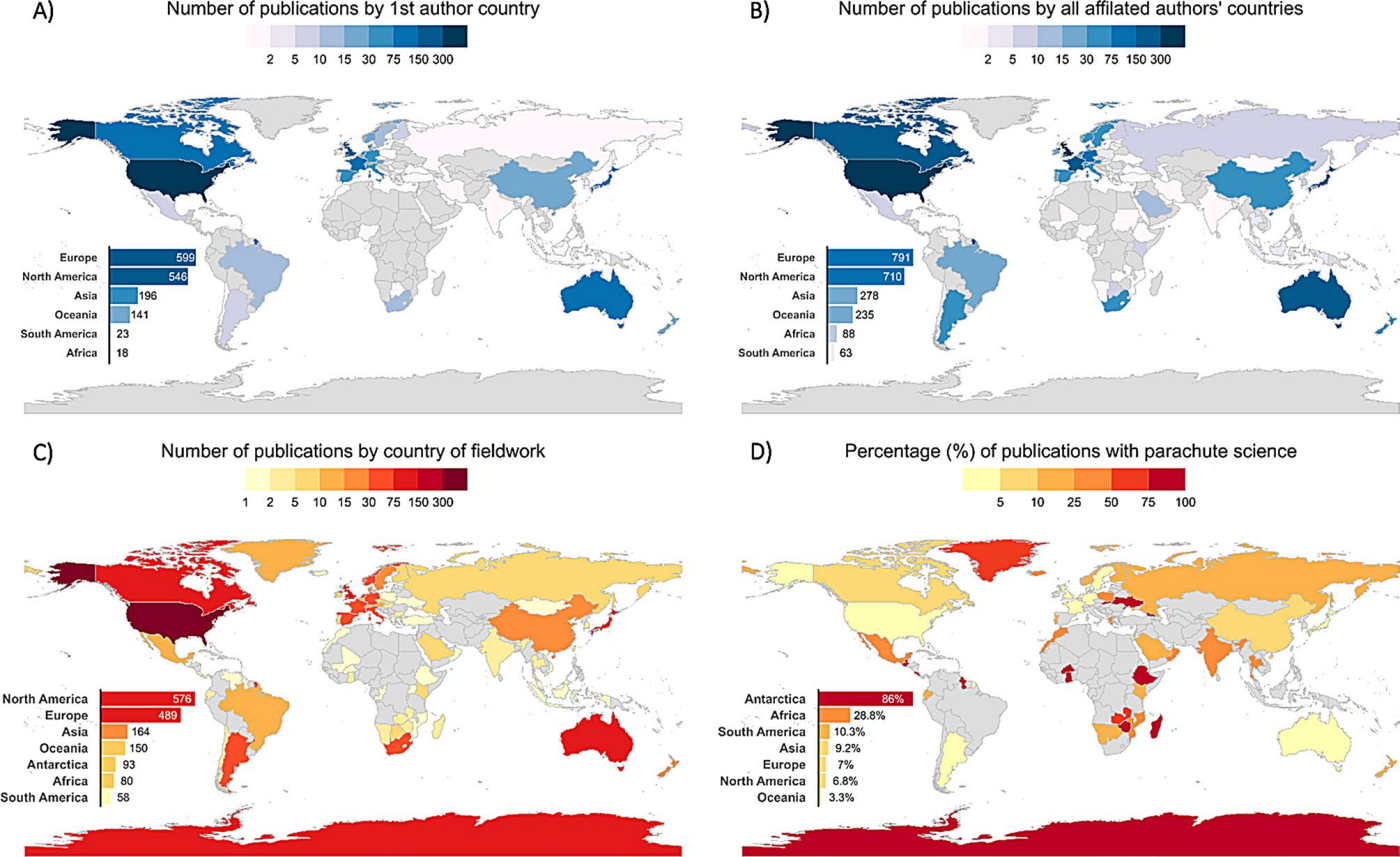
Geographic analyses of accelerometry literature. (**A**) Map showing the number of publications by country based on first author’s affiliation. (**B**) Map showing the number of publications by country considering all listed authors affiliation. (**C**) Map showing the number of publications by country where data were collected. Darker colours correspond to countries with greater number of publications. (**D**) Map illustrating the geographic occurrence of parachute science, defined as publications with no listed authors were affiliated with the country where data were collected. Darker red indicates countries with a high proportion of parachute science relative to the total number of publications for the given country. Bar plots to the bottom left of each map represent the total number of publications where science was published (**A** & **B**), data collected (**C**) or proportion of publications with parachute science (**D**) by geographic region

An important consideration to address is the language bias of this review (we only surveyed English language publications), which missed publications in other languages and may have missed important cultural contexts of some studies (e.g. in traditional farming methods [261]). First authors were affiliated with 52 countries (Fig. 8A), yet over half of publications (59%, *n* = 894) came from only five nations: USA (*n* = 402), Japan (*n* = 149), Canada (*n* = 132), Australia (*n* = 119) and the UK (*n* = 92). Regionally, Europe had the most publications (*n* = 599), followed by North America (*n* = 546), Asia (*n* = 196), Oceania (*n* = 141), South America (*n* = 23) and Africa (*n* = 18). Less than 6% (*n* = 87) of studies were led by first authors affiliated with institutions in Africa, South America and Asia (excluding Japan, as they are the second highest contributor to accelerometry research) combined, and these first authors were from only 19 countries (Fig. 8A). In total, 82 countries were represented across all authorships, but 51% of publications were from European authors, and 46% North American authors. We note that the present review is conducted by authors all based in Europe – we would hope accelerometry research is democratised to the extent that future reviews are not.

Accelerometry research can be prohibitively expensive, due to device cost, deployment logistics, and data infrastructure and analysis, perhaps creating barriers for research in low-income regions. The global distribution of publications correlates with national GDP (gross domestic product), underscoring how economic inequality influences access to and participation in technologically advanced research. Similar trends are apparent in where accelerometry data are collected. While data collection spanned 98 countries and regions (Fig. 8C), four of the top five publishing countries were also the primary sites for data collection: the USA (27%), Australia (8%), Canada (8%), and the UK (7%). The fifth most common location for tag deployment was Antarctica and the sub-Antarctic region (6%). In contrast, data collection was noticeably sparse in global biodiversity hotspots such as South America and Africa, with limited research outside southern African nations. Limited data collection occurred across the Caribbean, parts of the Middle East, Central Asia, Southeast Asia, the Pacific Islands, the Balkan and Baltic states (Fig. 8C). These geographic gaps often coincided with regions of lower GDP (gross domestic product), GDI (gross domestic income) [262] and Global Peace Index [263]. In addition to economic limitations, other potential barriers include restrictions on the import of research equipment, difficulties in obtaining permits, information technology obstacles, and logistical challenges related to safety, infrastructure deficits or political stability [264, 265].

**Fig. 9:**
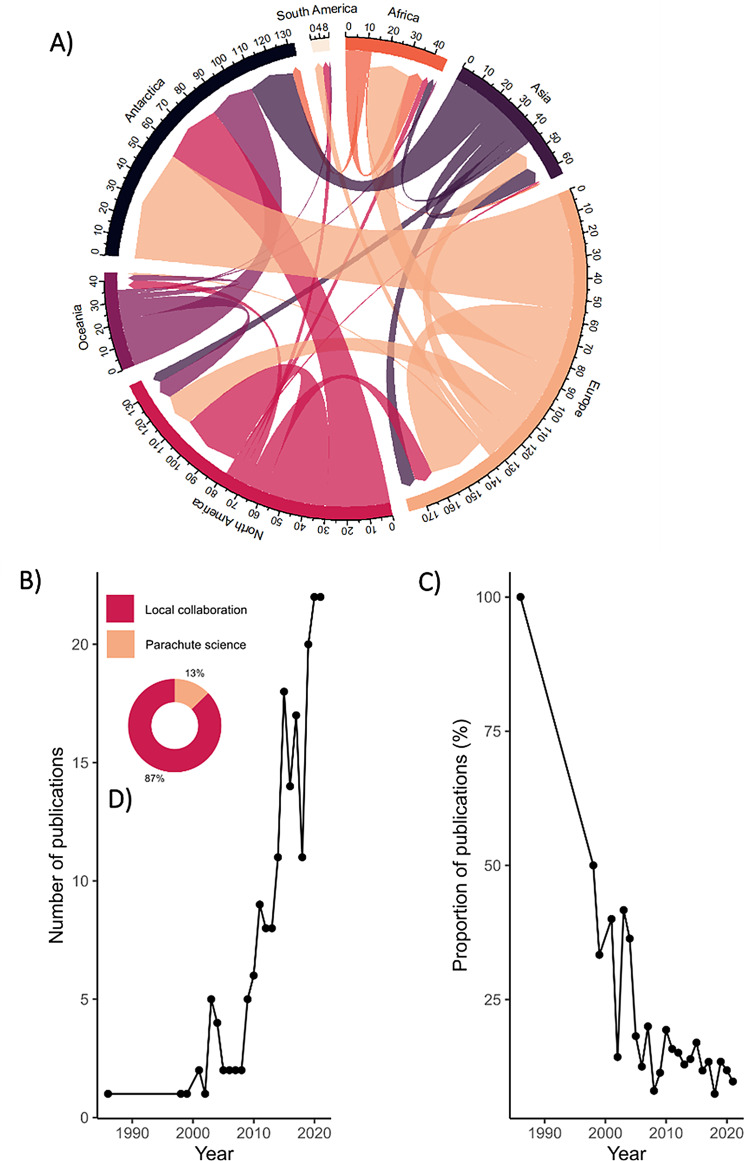
Trends and patterns of in parachute science in accelerometry research. (**A**) Chord diagram illustrating regional patterns of parachute science. Arrows indicate the direction of parachute science, pointing from the publishing region (based on author affiliation) to the region where data were collected. For example, as no authors are affiliated with the Antarctic region, all arrows point towards Antarctica. Arrow thickness represents the number of publications from one region collecting data in another. (**B**) Time series of the number of publications with parachute science per year. (**C**) Time series of the annual proportion of publications with parachute science relative to the total number of studies published per year. (**D**) Ring plot showing the overall proportion of studies where data were collected locally (i.e. at least one listed author was affiliated with the country where data were collected, shown in red) vs those exhibiting parachute science in orange

While most of the research is conducted locally (87%), that is to say, where at least one author is affiliated with the country where the data is being collected, 13% lacked local collaborators (Fig. 9D). Parachute science – where research is conducted in a country without local authorship – was recorded across all global regions, occurring in 55 of the 92 countries where data were collected, though to varying degree (Fig. 9D). It was least common in Oceania (3.3%) but occurred in over a quarter of studies conducted in Africa (28.8%) (Fig. 9D). European (*n* = 79) and North American countries (*n* = 49) were responsible for the highest number of cases (Fig. 9A). While the number of publications with parachute science has risen over time (Fig. 8B), its proportion has declined, averaging 13% since 2015 and dropping to approximately 9% in 2021 (Fig. 9C) [264].

These patterns closely mirror those in broader conservation and movement ecology literature where studies in Africa, Asia, and Latin America show substantially higher rates of research without local authorship (2–14%) compared to Europe, North America, and Oceania (0–2%) [266–268]. Despite increasing awareness of these issues, Shaw et al. [267] found no significant reduction in parachute science in movement ecology between 2009 and 2020, though our data suggest some encouraging change in accelerometry research. These results highlight the need for more inclusive research, with acknowledgement of data ownership or local expertise to be better recognised [126, 269]. As Asase et al. [270] emphasise, transitioning from ‘parachute science’ to ‘global science’ requires deep, permanent collaborations where researchers from all regions play collaborative rather than subordinate roles in posing questions, analysing data, and interpreting results. There is scope for additional capacity building and knowledge exchange of technical and analytical skills [264], as well as structural changes in funding and institutional support that enable equitable partnerships [270].

### Cross-disciplinary applications and knowledge transfer

Research on animals in managed care has long contributed to accelerometry studies, particularly in calibrating proxies for energy expenditure before field deployments [139, 241, 271], or tracking disease progression relevant to both veterinary [93, 272] and biomedical contexts [110]. Captive animals have also been used to develop and train behavioural classification models, acting as surrogates before deployment on wild counterparts. For example, models trained on captive American beavers had 95% accuracy when applied to wild European beavers [151]. Transferability depends on the model [194], species traits and environment in which the data were collected. For instance, models trained using data from a domestic dog successfully identifying three behavioural modes in seven other quadrupedal species [224]. However, models trained on phylogenetically-similar captive pygmy goats predicted alpine ibex behaviour with only 55% accuracy [225], highlighting limitations of even closely related surrogate species. Despite these challenges, model calibration using surrogates may provide valuable insight for cryptic, endangered or rare species, where direct behavioural validation is limited [224, 225].

In agriculture, accelerometers are used in PLF, offering real-time insights into health, welfare and productivity. Methods developed for terrestrial livestock are now being transferred to aquaculture, where adoption has been historically slower. Accelerometry is increasingly being applied to farmed fish and invertebrates to detect changes in behaviour associated with feeding, selective breeding, stocking density, parasitism, and energy use [78, 79, 81, 271, 273]. These can inform management practices such as habitat enrichment, transportation, and minimising human disturbance, optimising feeding times, and the change in behaviour with different feed types [80, 274–277]. Accelerometry may be particularly valuable in turbid, crowded or low-light aquatic conditions where visual monitoring techniques are intractable. Accelerometers that can remotely transmit summary metrics such as activity levels, a feature well-established in PLF systems, are continually gaining traction in aquaculture [58], exemplifying successful cross-disciplinary technology transfer.

Welfare-focused accelerometry studies have been predominantly used on mammals, particularly domesticated or commercially important species [83, 86, 88]. Accelerometers may be more widely used to continuously track changes in affective states over time [50], for example in zoos and aquaria [88, 90, 91] to assess housing conditions, enrichment regimes, food supplementation and treatment efficacy. In wild animals, accelerometers paired with other sensors (e.g. temperature, heart rate, GPS) can provide context to behaviours and help assess how conditions such as climatic events, anthropogenic disturbances and management practices, affect welfare [53, 278–282]. However, the processes of capture, handling, and accelerometer attachment, particularly on wild animals, can themselves compromise welfare. Therefore, ongoing refinement of design and deployment practices will be essential to meaningfully advance welfare science [176].

### Technological advancements and future directions

Some of the biggest limitations faced by accelerometry studies relate to data storage and battery longevity whilst minimising tag weight – devices tend to store acceleration data on up to three axes many times a second and usually do not remotely transmit it. Battery consumption scales with sampling frequency [283]; doubling the sampling rate from 20 Hz to 40 Hz can require doubling or more the battery capacity, and hence the device weight [284]. Comparatively, while a 0.5 *g* VHF tag may track an animal for a month, achieving a similar deployment duration with high frequency accelerometry required batteries 10–100 times heavier [284]. While modern accelerometers are relatively power efficient (5–10 mA at 40 Hz) compared to GPS devices (30–50 mA at 1 Hz [285]), incorporating additional sensors substantially increases power demands. Recent advances in low-power design have achieved increased efficiency. For example, an audio-inertial logger using MEMS sensors and optimised firmware consumed just 5.3 mW, enabling 900 hours of continuous multi-sensor recording (8 kHz audio, 50 Hz accelerometer, 10 Hz magnetometer) on a compact battery [286].

Beyond optimising power consumption, alternative energy sources offer complimentary solutions [283]. Solar-powered devices enable continuous operation in favourable conditions [153, 287, 288], though this may exclude aquatic and nocturnal species. Energy harvesting from animal movement via piezoelectric materials presents a more universal approach, with substantial advances in this rapidly growing field [289], offering the potential for endogenous power generation across diverse uses [283]. Despite these advances, most accelerometry devices still must be physically recovered to access data. This not only risks data loss (if devices are not recovered) but also requires recapture or repeated handling of animals, with associated logistical and ethical implications.

To overcome data loss, particularly in very mobile species or remote or aquatic environments where tag recovery is unreliable, data transmission using summary metrics has been developed, such as those used in pop-off satellite archival tags (PSATs). For instance, Nielsen et al. [290] used on-board processing of tilt data to calculate survival metrics for bycaught Pacific halibut, transmitted via the Argos satellite system. Similarly, Skubel et al. [291] developed a programmable activity time-series metric, while Cox et al. [218] and Heerah et al. [292] used prey capture abstraction for southern elephant seals and Weddell seals respectively. In aquatic systems, acoustic accelerometer transmitters have increasingly been used, preprocessing signals on-board by filtering the static component and calculating the root-mean square of the dynamic acceleration, transmitted via acoustic pings [58, 274, 290, 291].

Where transmission is not possible, several strategies have emerged to compress data directly on the device. Archival devices are often restricted by limited storage, constraining either sampling frequency or deployment duration. This can be mitigated through asynchronous sampling using pre-defined sampling windows to enable longer deployments [155, 163–166]. On-board ML classification also offers a particularly promising solution. Le Roux et al. [221] demonstrated a 27-fold reduction in energy consumption and almost 500-fold reduction in memory usage when behavioural classification was performed on-board rather than transmitting raw data. Approaches like knowledge distillation, which compresses complex network models into smaller, more efficient versions, could facilitate future on-chip behavioural classification [82]. For instance, Tanigaki et al. [293] developed an AI-enabled biologger that could classify outlier (or ‘rare’) behaviours on streaked shearwaters, triggering power-intensive video cameras only when rare behaviours were detected. Summary statistics can also compress raw accelerometry data prior to storage [221, 294]. Bäckman et al. [166] used intermittent sampling with threshold-based models to identify flight in red-backed shrikes, enabling year-long tracking of small birds previously unachievable due to size constraints.

Acceleration thresholds can support real-time alert systems across multiple fields, facilitating timely intervention during key welfare events such as hypoxia, disease onset and even spawning [295, 296]. In agriculture, ML applied to accelerometry data predicts parturition in dairy cows, triggering birthing alarms and reducing calf mortality [297], while similar methods detect pre-farrowing behaviour in sows to optimise crating timing [298]. In wildlife management, devices can notify managers when movement ceases, potentially signalling mortality [287], while alerting systems could extend to biomedical, veterinary and aquaculture settings, where behavioural changes may reflect illness or stress [80, 296, 299]. However, practical barriers remain. High computational demands for edge computing (a distributed computing model that processes data closer to the generation source, reducing latency and improving real-time processing [300]) and costs of accelerometers can limit uptake, particularly as aquaculture is predominantly conducted at small- to medium-scale settings in developing countries in contrast to large scale terrestrial farming largely dominated by high earning countries [80, 86].

Acceleration-triggered sensors also reduce data volume by activating other sensors only during targeted behaviours. Jaw- or head-mounted cameras on northern elephant seals were programmed to record only when acceleration indicated foraging [301, 302], while activity thresholds can modulate GPS fixes, dynamically adjusting the sampling rate according to behaviour [303, 304]. Park et al. [305] demonstrated a fully integrated system where behavioural classification jointly managed GPS and high-definition video through on-board logic to autonomously decide when and how to record each modality in a remote animal-borne network monitoring system.

While summarised metrics and on-board classification can greatly extend deployment duration and reduce handling, they introduce important trade-offs. The main risk is missing rare or subtle behaviours due to algorithm sensitivity limitations [293], pre-processing choices or classification errors [218], potentially underestimating time-activity budgets [306]. These risks are further compounded when models trained under controlled conditions are deployed in complex natural environments [194]. Careful validation of compressed recording strategies is required, considering species-specific behaviour, age class, attachment method and environmental conditions [307]. Chen et al. [308] developed a software-based simulation allowing users to virtually test data collection strategies using synchronised video data, enabling cost-effective validation prior to deployment.

Addressing these challenges in animal-based accelerometry research requires learning from sectors that have already mastered the processing and visualisation of complex, multi-channel movement data. The aerospace industry routinely processes and visualises tens of simultaneous data channels in real-time for operational flight management and training simulators, presenting parallel challenges to multi-sensor biologging tags. Similarly, the consumer wearable device industry (smartphones, smartwatches and activity trackers) has developed sophisticated, real-time analysis and compelling visualisations of GPS and accelerometry data, transforming complex daily movement patterns into accessible insights, such as gait analyses, activity performance metrics and early detection of degenerative conditions through continuous movement monitoring [309, 310]. Visualisation of movement data for fitness users and clinicians, as well as user preference studies have become major research priorities in these fields [309–313]. In contrast, similar research has lagged behind in animal accelerometry. Initiatives such as middlewear for real-time wearable data analysis [314], RAPIDS for reproducible data streams [315] and the Wearables for Heath Toolkit for centralised data management and visualisation [313] highlight the potential for developing similar tools for animal accelerometry, improving data interpretation, scientific communication, and accessibility to non-specialists.

The ongoing refinement of accelerometry devices, particularly on-board processing in off-the-shelf tags, presents promising avenues for broadening accessibility and application. By summarising activity and/or classifying behaviour directly on the device, the need for complex post-processing is reduced, making data more interpretable to end-users without specialist expertise. This analytical simplicity, combined with lower device costs compared to multi-sensor alternatives, may help bridge current geographic and taxonomic gaps by enabling broader global deployment across diverse species. Commercially available accelerometers such as the Actical tag (Philips Respironics) initially developed for livestock and clinical use, exemplify this accessibility, monitoring welfare in poultry [316, 317], tracking response to treatment in domestic animals [318–320], assessing husbandry methods in cows [321], investigating Parkinson’s disease in primate models [322] and juvenile obesity in porcine models [323]. Importantly, their successful deployment in ecological studies demonstrates versatility across environments and research contexts. From tracking behavioural shifts in aardvarks during droughts [324], to documenting thermoregulatory responses in Arabian oryx [325], and evaluating king penguin responses to drones [326], these devices offer pragmatic solutions for broad ecological questions particularly in understudied taxa or where research funding is limited.

### Open science and collaborative approaches

Open data repositories for biologging data, such as MoveBank [327] and WILDLABS Inventory, enable researchers to share data and tools across disciplines. These repositories provide resources for the community and stakeholders [191], and facilitate large-scale, multi-species projects. However, community trust, consistent metadata standards, uniform data formatting and clear attribution protocols are essential [124, 328–330]. There is no similar repository of acceleration data, but there are a number of open-source software tools, predominantly available in R. There are other, online, tools such as AcceleRater (which can be used for supervised learning of behaviour [331], Framework4 (used to model trajectory, behaviour and energy expenditure [285]), and Semantic Annotation and Activity Recognition (SAAR, which supports storing, visualising, annotating, and automatic recognition of accelerometer data with associated video [332]). For deep learning, frameworks like LiteRT (formerly TensorFlow Lite) enable behavioural inference using pretrained models [333], while benchmarking platforms such as BEBE (Bio-logger Ethogram Benchmark) provide standardised datasets and evaluation metrics for comparing ML analyses [334].

Beyond software accessibility, open-electronics initiatives are addressing the hardware barrier that limit accelerometry research globally. Low-cost, customisable sensors offer an affordable alternative to commercial tags [335, 336], with initiatives such as Mataki attempting to democratise sensor technology. While such initiatives are important, their sustainability requires ongoing (and time consuming) commitment. For example, component specifications and chip availability constantly change, meaning that individual board designs inevitably have limited lifespans. Democratisation of sensor technology will be valuable where prohibitively expensive devices have concentrated accelerometry studies primarily in the high earning countries. The success of open approaches also relies on fostering interdisciplinary collaboration [283], including greater exchange in methodology, expertise, technical skills and analytical approaches, which will help fully harness the potential of accelerometers and maximise the value of rich datasets. Realising this potential requires not only technical infrastructure but also structural support from funding agencies. Incentivising collaborative grants that span traditional disciplinary boundaries and require data sharing agreements could reduce redundant tagging efforts, maximise resource efficiency, and accelerate progress across the field while minimising cumulative impacts on animal welfare. 

## Electronic supplementary material

Below is the link to the electronic supplementary material.

Supplementary material 1

## Data Availability

The datasets generated and analysed during the current study are available in the Figshare repository: 10.6084/m9.figshare.31332391.

## Abbreviations

AI: Artificial intelligence
BEBE: Bio-logger Ethogram Benchmark
CPR: Cardiopulmonary Resuscitation
DBA: Dynamic Body Acceleration
DLW: Doubly Labelled Water
ECG: Electrocardiogram
EEH: Electroencephalogram
EMG: Electromyogram
EOG: Electrooculogram
GDI: Gross Domestic Income
GDP: Gross Domestic Product
IMU: Inertial Measurement Unit
IOT: Internet of Things
LLM: Large Language Model
MEMS: Micro-Electro Mechanical Systems
ML: Machine Learning
ODBA: Overall Dynamic Body Acceleration
PSAT: Pop-off Satellite Archival Tags
SAAR: Semantic Annotation and Activity Recognition
UHF: Ultra High Frequency
VeDBA: Vectorial Dynamic Body Acceleration
VHF: Very High Frequency
