# Supplementary material for: Six decades of animal accelerometry: trends, applications, and future directions
Source: Mov Ecol. 2026 Apr 2;14:23. doi: 10.1186/s40462-026-00641-1 (PMC13045147; doi:10.1186/s40462-026-00641-1)
Supplement: Supplementary file 1 — Supplementary material 1 [file 40462_2026_641_MOESM1_ESM.docx]

**Supplementary Materials**

**Supplementary Methods**

Query design and eligibility criteria

All searches were conducted in February 2022, including records published between 1900 and 2021 inclusive.

A total of 9,933 records were identified from an initial Google Scholar search using the reference manager tool Publish or Perish from the following combinations of terms: Accelerometer + animal; Accelerometer + animals; Accelerometer + wildlife; Accelerometer + mammal; Accelerometer + bird; Accelerometer + fish; Accelerometer + reptile; Accelerometer + amphibian; Accelerometer + invertebrate. A further literature search was conducted using Web of Science with the following more comprehensive query:

ALL=((accelerometer* OR accelerometry OR biologger* OR bio-logger* OR biologging OR bio-logging) AND (bird* OR mammal* OR fish* OR reptile* OR amphibian* OR invertebrate* OR animal* OR wildlife))

The Web of Science query resulted in an additional 2411 records, leading to a total of 12,343 records. Duplicate entries, grey literature, book chapters, reviews, conference submissions, articles not published in English, and those without accessible full texts were excluded. Abstracts were then assessed for relevance, and publications unrelated to the research focus (e.g. studies on accelerometers used in amphibious warfare, or in acoustic research using particle motion) were removed. This screening process resulted in 4285 articles for full text assessment. To be included in the review, however, studies had to involve accelerometers attached to live animals, with accelerometry data actively used in the article, so a further 2672 records were excluded, along with another 89 articles that were not directly relevant. A total of 1524 articles proceeded to the qualitative synthesis, during which 32 metrics were collected (see section below) (Figure 2, Supplementary Table 1).

Metrics collected

The following metrics were collected from each publications meeting the inclusion criteria (see also Supplementary Tables 1-3): publication title, first author name, year of publication, country affiliated with first author, total number of authors, countries affiliated with all authors, keywords, tag manufacturer, tag model, recording frequency, number of successfully recovered and functioning tags, number of axes the accelerometer used, attachment location, top two research topics, country of data collection, animal system (not mutually exclusive: Aerial, Terrestrial, Marine, Aquatic), animal class (mammal, bird, fish, reptile, amphibian, invertebrate), species, status (captive, wild), domestication (domestic, not domestic), sample size, sex, life stage (adult, sub-adult, juvenile), tracking duration, parameters of accelerometry used, sampling frequency used for analysis, additional sensors used (e.g. temperature, pressure, ECG), software for analysis, machine learning approach (supervised, semi-supervised, unsupervised), machine learning algorithms (e.g. Random Forest, Hidden Markov Model, Artificial Neural Network) and publication summary. When information was missing or unclear, it was listed as NA to avoid ambiguity. All publications and documented information were evaluated by a single reviewer (JR or HW).

Sampling frequency and tracking duration

Each study’s sampling frequency was categorised into sampling bins for analysis (<1 Hz, 1-10 Hz, 10-50 Hz, 50-100 Hz, > 100 Hz and > 1000 Hz). Where possible, the mean and the range of tracking durations were recorded for each publication and classified into categories (< 1 min, < 1 hour, <1 day, < 1 week, < 1 month, < 6 months, < 1 year, > 1 year) based on the mean, or on the range when no mean was available.

**Supplementary Table 1:** Table of all the studies and associated metrics analysed within this review. Each row represents an individual study. The variables include the publication title, first author, year of publication, country affiliated with the first author, region (continent) affiliated with the first author, number of authors listed on the publication, countries affiliated with all authors listed on the publication, countries where the data were collected, regions where the data were collected, tag model and manufacturer, sampling frequency bracket, number of accelerometer axes, deployment duration, taxon, sample size by taxon and total sample size of the publication (tags successfully recovered and employed in the study), species, status (whether animals were studied in captivity or in the wild), main research topics and subtopic, the use of additional sensors other than accelerometers, the software used for analysis (only the eight most used softwares are included), and what machine learning approach and algorithm were used for behavioural classification.

The table is hosted on the Figshare repository, <https://doi.org/10.6084/m9.figshare.31332391> **Supplementary Table 2**: Grouping of research topics by broader category. Research topics listed are non-exhaustive

| **Research categories** | **Original Research topics** |
| --- | --- |
| Behavioural studies | Foraging, Sleep, Reproductive behaviour, Activity, Behavioural classification, Time activity budget, Vocalisation, Communication, Parental care, Behaviour, Territoriality |
| Energy expenditure and Physiology | Energy expenditure, Cardiac activity, Metabolic rate, Physiology, Stress physiology, Thermogenesis, Thermoregulation, Ventilation, Respiration, Body condition |
| Environmental & Ecological studies | Predation, Conspecific interactions, Predator-prey interactions, Competition, Parasitism, Spatial ecology, Hierarchy, Niche partitioning, Environmental conditions, Seasonal variation, Sex differences, Age class differences, Oceanography, Ontogeny |
| Human impact | Anthropogenic disturbance, Human-wildlife conflict, Bycatch, Pollution (noise, chemical, light...) |
| Movement and Biomechanics | Dive behaviour, Swimming behaviour, Flight behaviour, Locomotion, Migration, Kinematics, Biomechanics, Buoyancy, Drag, Navigation, Aerodynamics, Hydrodynamics, Movement ecology |
| Neurosciences and Medicine | Medical sciences, Neurosciences, Trauma response, Brain injury, Hearing, Vision, Surgery method, Cognition, Tremors, Bone vibration, Memory, Muscle function, Lateralisation |
| Technology and Methods | Sensor development, Sensor performance, Sensor comparison, Method development, Method validation, Data visualisation, Machine Learning, Algorithm development, Algorithm performance, Metric development, Dead reckoning, Tag attachment comparison, Energy harvesting, Surrogate species use, sampling frequency comparison, Calibration |
| Welfare, Health and Husbandry | Treatment response, Treatment type, Welfare, Capture effect, Tag effect, Post-release behaviour/mortality, Lameness, Disease detection, Disease, Injury, Animal husbandry, Habitat enrichment, Housing condition, Heat stress, Reintroduction, Food supplementation, Mortality, Survival |

**Supplementary Table 3**: Grouping of machine learning algorithms by broader categories

| Algorithm category | Machine learning algorithms |
| --- | --- |
| Bayesian method | Bayesian Markov Model, Hidden Markov Model, Naive Bayes |
| Clustering | K-means clustering, Agglomerative Hierarchical Clustering, Fuzzy C-Means, Kohonen super Self Organising Map algorithm, Self-Organising Map Network Algorithms, Superparamagnetic Clustering Algorithm |
| Decision tree | Adaptive Boosting, Classification and Regression Tree, Decision Tree, Extreme Gradient Boosting, Gradient Descent Multi-Class Boosting, Gradient Boosting, LogitBoost-with-trees, Multiclass Adaptive Boosting Meta-classifier, Random Forest, Synthetic Minority Boosting |
| Dimensionality reduction | Principal Component Analysis |
| Discriminant analysis | Discriminant Analysis, Canonical Discriminant Analysis, Linear Discriminant Analysis, Quadratic Discriminant Analysis |
| Distance-based method | K-nearest neighbour, Support Vector Machine |
| Ensemble model | Ensemble Classification Approaches Bagging, Random Subspace, Super Learner, Gradient Multi-class Stochastic Boosting, Multi-class Stochastic Boosting, Multi-stage Adaptive Boosting |
| Neural Networks | Neural Network, Artificial Neural Network, Back-Propagation Neural Network, Convolutional Neural Network, Feedforward Neural Network, Input Delay Neural Network, Model Averaged Neural Network, Multi-layer Perceptron Neural Network |
| Regression method | Generalised Linear Elastic Net, Generalised Linear Model, Linear Classifier, Linear Regression, Logistic Regression, Multivariate Adaptive Regression Spline, Partial Least Square Regression |
| Other | Adaptive Neuro-Fuzzy Inference System classifier, Boolean-based Classification Algorithm, Expected Maximisation Algorithm, Genetic Programming Evolutionary Machine Learning, ASSEMBLE Semi-supervised Machine Learning |

**Supplementary Table 4**: Table of species on which accelerometers have been deployed. The table includes the species class, whether it is categorised as domestic or not, the number of publications per species, and whether the studies were conducted on wild and/or captive individuals.

| Species | Taxon | Domesticated | Count | Status |
| --- | --- | --- | --- | --- |
| Aardvark | Mammal | Non_domestic | 2 | Wild |
| Adelie penguin | Bird | Non_domestic | 11 | Captive, Wild |
| African buffalo | Mammal | Non_domestic | 1 | Wild |
| African cheetah | Mammal | Non_domestic | 10 | Captive, Wild |
| African elephant | Mammal | Non_domestic | 6 | Captive, Wild |
| African green monkey | Mammal | Non_domestic | 1 | Captive |
| African leopard | Mammal | Non_domestic | 2 | Wild |
| African lion | Mammal | Non_domestic | 9 | Wild |
| African penguin | Bird | Non_domestic | 1 | Wild |
| African wild dog | Mammal | Non_domestic | 4 | Captive, Wild |
| Aldabra giant tortoise | Reptile | Non_domestic | 2 | Captive, Wild |
| Alpine chamois | Mammal | Non_domestic | 2 | Wild |
| Alpine chipmunk | Mammal | Non_domestic | 2 | Captive, Wild |
| Alpine ibex | Mammal | Non_domestic | 5 | Captive, Wild |
| Amazonian manatee | Mammal | Non_domestic | 1 | Wild |
| American alligator | Reptile | Non_domestic | 2 | Captive, Wild |
| American horseshoe crab | Invertebrate | Non_domestic | 5 | Captive, Wild |
| American lobster | Invertebrate | Non_domestic | 4 | Captive, Wild |
| Andean condor | Bird | Non_domestic | 3 | Wild |
| Antarctic fur seal | Mammal | Non_domestic | 9 | Captive, Wild |
| Antillean manatee | Mammal | Non_domestic | 1 | Captive |
| Arabian oryx | Mammal | Non_domestic | 6 | Wild |
| Arabian sand gazelle | Mammal | Non_domestic | 1 | Captive |
| Arapaima | Fish | Non_domestic | 1 | Wild |
| Arctic charr | Fish | Non_domestic | 1 | Captive, Wild |
| Arctic fox | Mammal | Non_domestic | 1 | Wild |
| Arctic ground squirrel | Mammal | Non_domestic | 3 | Wild |
| Argentine black and white tegu | Reptile | Non_domestic | 1 | Captive |
| Argentine sea bass | Fish | Non_domestic | 2 | Captive, Wild |
| Argentinean tuco-tuco | Mammal | Non_domestic | 2 | Captive |
| Asian elephant | Mammal | Non_domestic | 3 | Captive |
| Astatotilapia burtoni | Fish | Non_domestic | 1 | Captive |
| Atlantic bluefin tuna | Fish | Non_domestic | 2 | Wild |
| Atlantic cod | Fish | Non_domestic | 5 | Captive, Wild |
| Atlantic Goliath grouper | Fish | Non_domestic | 1 | Wild |
| Atlantic halibut | Fish | Non_domestic | 1 | Wild |
| Atlantic salmon | Fish | Non_domestic | 7 | Captive, Wild |
| Australasian gannet | Bird | Non_domestic | 4 | Wild |
| Australian fur seal | Mammal | Non_domestic | 7 | Captive, Wild |
| Australian magpie | Bird | Non_domestic | 1 | Wild |
| Australian sea lion | Mammal | Non_domestic | 4 | Captive |
| Baikal seal | Mammal | Non_domestic | 4 | Captive, Wild |
| Bald notothen | Fish | Non_domestic | 1 | Captive |
| Banded morwong | Fish | Non_domestic | 1 | Captive, Wild |
| Bar-headed goose | Bird | Non_domestic | 1 | Wild |
| Barnacle goose | Bird | Non_domestic | 4 | Captive, Wild |
| Basking shark | Fish | Non_domestic | 2 | Wild |
| Baudin's black cockatoo | Bird | Non_domestic | 1 | Captive, Wild |
| Bearded vulture | Bird | Non_domestic | 1 | Wild |
| Bengal tiger | Mammal | Non_domestic | 1 | Captive |
| Bewick's swan | Bird | Non_domestic | 3 | Captive, Wild |
| Big brown bat | Mammal | Non_domestic | 1 | Captive |
| Black bear | Mammal | Non_domestic | 7 | Captive, Wild |
| Black ghost knife fish | Fish | Non_domestic | 1 | Captive |
| Black kite | Bird | Non_domestic | 1 | Wild |
| Black olive ridley turtle | Reptile | Non_domestic | 1 | Captive |
| Black rhinoceros | Mammal | Non_domestic | 3 | Captive |
| Black swan | Bird | Non_domestic | 1 | Captive |
| Black-browed albatross | Bird | Non_domestic | 3 | Wild |
| Black-footed albatross | Bird | Non_domestic | 1 | Wild |
| Black-legged kittiwake | Bird | Non_domestic | 9 | Wild |
| Black-tailed deer | Mammal | Non_domestic | 1 | Wild |
| Black-tailed gull | Bird | Non_domestic | 1 | Wild |
| Blacktip reef shark | Fish | Non_domestic | 2 | Wild |
| Blacktip shark | Fish | Non_domestic | 6 | Captive, Wild |
| Blainville's beaked whale | Mammal | Non_domestic | 6 | Wild |
| Blue shark | Fish | Non_domestic | 1 | Wild |
| Blue whale | Mammal | Non_domestic | 17 | Wild |
| Blue wildebeest | Mammal | Non_domestic | 5 | Captive, Wild |
| Bluefish | Fish | Non_domestic | 5 | Captive |
| Blue-footed boobie | Bird | Non_domestic | 1 | Wild |
| Blue-lipped sea krait | Reptile | Non_domestic | 1 | Captive |
| Bluespotted flathead | Fish | Non_domestic | 1 | Wild |
| Bluntnose sixgill shark | Fish | Non_domestic | 2 | Wild |
| Bobcat | Mammal | Non_domestic | 3 | Captive, Wild |
| Bocaccio rockfish | Fish | Non_domestic | 1 | Wild |
| Bonefish | Fish | Non_domestic | 5 | Captive, Wild |
| Bottlenose dolphin | Mammal | Non_domestic | 11 | Captive |
| Bowhead whale | Mammal | Non_domestic | 2 | Wild |
| Brazilian tapir | Mammal | Non_domestic | 1 | Captive |
| Brent goose | Bird | Non_domestic | 2 | Wild |
| Brown bear | Mammal | Non_domestic | 3 | Captive, Wild |
| Brown boobie | Bird | Non_domestic | 3 | Wild |
| Brown pelican | Bird | Non_domestic | 1 | Wild |
| Brunnich's guillemot | Bird | Non_domestic | 2 | Wild |
| Bryde's whale | Mammal | Non_domestic | 5 | Wild |
| Bull shark | Fish | Non_domestic | 4 | Captive, Wild |
| Burbot | Fish | Non_domestic | 1 | Wild |
| Burmese pyhton | Reptile | Non_domestic | 1 | Wild |
| California mussel | Invertebrate | Non_domestic | 2 | Wild |
| California sea lion | Mammal | Non_domestic | 5 | Captive, Wild |
| Call duck | Bird | Non_domestic | 1 | Captive |
| Camel | Mammal | Non_domestic | 2 | Captive, Wild |
| Canada goose | Bird | Non_domestic | 2 | Captive, Wild |
| Canada lynx | Mammal | Non_domestic | 1 | Wild |
| Cane toad | Amphibian | Non_domestic | 1 | Captive |
| Cape cormorant | Bird | Non_domestic | 1 | Wild |
| Cape gannet | Bird | Non_domestic | 2 | Wild |
| Capercaillie | Bird | Non_domestic | 1 | Wild |
| Caribbean reef shark | Fish | Non_domestic | 1 | Wild |
| Caribbean spiny lobster | Invertebrate | Non_domestic | 2 | Captive, Wild |
| Caribou | Mammal | Non_domestic | 3 | Captive, Wild |
| Carnaby's black cockatoo | Bird | Non_domestic | 1 | Captive, Wild |
| Cat | Mammal | Domestic | 19 | Captive |
| Chacma baboon | Mammal | Non_domestic | 5 | Captive, Wild |
| Channel catfish | Fish | Non_domestic | 1 | Wild |
| Chestnut-mandibled toucan | Bird | Non_domestic | 1 | Wild |
| Chicken | Bird | Domestic | 19 | Captive |
| Chinese sturgeon | Fish | Non_domestic | 2 | Wild |
| Chinook salmon | Fish | Non_domestic | 1 | Captive |
| Chinstrap penguin | Bird | Non_domestic | 5 | Wild |
| Chukar partridge | Bird | Domestic | 3 | Captive |
| Chum salmon | Fish | Non_domestic | 2 | Wild |
| Cinereous vulture | Bird | Non_domestic | 1 | Wild |
| Cobia | Fish | Non_domestic | 1 | Captive |
| Cockatiel | Bird | Non_domestic | 1 | Captive |
| Common cuttlefish | Invertebrate | Non_domestic | 1 | Captive |
| Common eland | Mammal | Non_domestic | 1 | Wild |
| Common guillemot | Bird | Non_domestic | 3 | Wild |
| Common murre | Bird | Non_domestic | 1 | Wild |
| Common swift | Bird | Non_domestic | 1 | Wild |
| Cow | Mammal | Domestic | 235 | Captive |
| Cowcod Rockfish | Fish | Non_domestic | 1 | Wild |
| Coyote | Mammal | Non_domestic | 3 | Wild |
| Coypu | Mammal | Non_domestic | 3 | Captive |
| Crab plover | Bird | Non_domestic | 1 | Wild |
| Crab-eating macaque | Mammal | Non_domestic | 4 | Captive |
| Cuvier's beaked whale | Mammal | Non_domestic | 5 | Wild |
| Dark-eyed Junco | Bird | Non_domestic | 1 | Captive |
| Deacon rockfish | Fish | Non_domestic | 1 | Wild |
| Death's head cockroach | Invertebrate | Non_domestic | 2 | Captive |
| Dingo | Mammal | Non_domestic | 4 | Captive, Wild |
| Dog | Mammal | Domestic | 87 | Captive |
| Dusky flathead | Fish | Non_domestic | 2 | Wild |
| Eastern box turtle | Reptile | Non_domestic | 1 | Wild |
| Eastern chipmunk | Mammal | Non_domestic | 1 | Wild |
| Eastern elliptio | Invertebrate | Non_domestic | 1 | Captive |
| Eastern grey kangaroo | Mammal | Non_domestic | 2 | Captive |
| Elegant crested tinamou | Bird | Non_domestic | 1 | Captive |
| Elegant sea snake | Reptile | Non_domestic | 2 | Captive, Wild |
| Emerald rockcod | Fish | Non_domestic | 1 | Captive |
| Emperor penguin | Bird | Non_domestic | 10 | Captive, Wild |
| Estuary stingray | Fish | Non_domestic | 1 | Wild |
| Eurasian beaver | Mammal | Non_domestic | 4 | Captive, Wild |
| Eurasian curlew | Bird | Non_domestic | 1 | Wild |
| Eurasian griffon vulture | Bird | Non_domestic | 10 | Captive, Wild |
| Eurasian hoopoe | Bird | Non_domestic | 1 | Wild |
| Eurasian lynx | Mammal | Non_domestic | 1 | Wild |
| Eurasian mountain reindeer | Mammal | Non_domestic | 1 | Wild |
| Eurasian oystercatcher | Bird | Non_domestic | 3 | Wild |
| European badger | Mammal | Non_domestic | 12 | Captive, Wild |
| European brown hare | Mammal | Non_domestic | 3 | Wild |
| European greenfinch | Bird | Non_domestic | 1 | Captive |
| European griffon vulture | Bird | Non_domestic | 1 | Wild |
| European hedgehog | Mammal | Non_domestic | 3 | Wild |
| European noctula bat | Mammal | Non_domestic | 1 | Captive |
| European pond turtle | Reptile | Non_domestic | 1 | Captive |
| European sea bass | Fish | Non_domestic | 4 | Captive |
| European sea bream | Fish | Non_domestic | 1 | Captive |
| European shag | Bird | Non_domestic | 7 | Wild |
| European spiny lobster | Invertebrate | Non_domestic | 1 | Captive |
| European starling | Bird | Non_domestic | 1 | Captive |
| European wildcat | Mammal | Non_domestic | 1 | Wild |
| Ezo abalone | Invertebrate | Non_domestic | 1 | Captive |
| Fallow deer | Mammal | Non_domestic | 1 | Wild |
| False death's head cockroach | Invertebrate | Non_domestic | 1 | Captive |
| False killer whale | Mammal | Non_domestic | 2 | Captive |
| Fin whale | Mammal | Non_domestic | 11 | Wild |
| Fiordland penguin | Bird | Non_domestic | 1 | Wild |
| Fisher | Mammal | Non_domestic | 6 | Wild |
| Fleurete's sportive lemur | Mammal | Non_domestic | 1 | Wild |
| Flying fox | Mammal | Non_domestic | 1 | Captive, Wild |
| Forest red-tailed black cockatoo | Bird | Non_domestic | 1 | Captive, Wild |
| Galapagos sea lion | Mammal | Non_domestic | 1 | Wild |
| Gemsbok | Mammal | Non_domestic | 2 | Wild |
| Gentoo penguin | Bird | Non_domestic | 5 | Wild |
| Geoffroy's cat | Mammal | Non_domestic | 1 | Captive |
| Geoffroy's spider monkey | Mammal | Non_domestic | 1 | Captive, Wild |
| Giant ant-eater | Mammal | Non_domestic | 1 | Captive |
| Giant armadillo | Mammal | Non_domestic | 1 | Wild |
| Giant Australian cuttlefish | Invertebrate | Non_domestic | 1 | Wild |
| Giant panda | Mammal | Non_domestic | 3 | Captive, Wild |
| Giant spider conch | Invertebrate | Non_domestic | 1 | Captive |
| Gilthead seabream | Fish | Non_domestic | 9 | Captive |
| Goat | Mammal | Domestic | 19 | Captive, Wild |
| Golden eagle | Bird | Non_domestic | 1 | Captive, Wild |
| Golden hamster | Mammal | Domestic | 2 | Captive |
| Golden lion tamarin | Mammal | Non_domestic | 1 | Wild |
| Golden perch | Fish | Non_domestic | 1 | Wild |
| Gray brocket deer | Mammal | Non_domestic | 1 | Wild |
| Gray whale | Mammal | Non_domestic | 2 | Wild |
| Great barracuda | Fish | Non_domestic | 1 | Wild |
| Great cormorant | Bird | Non_domestic | 2 | Captive, Wild |
| Great egret | Bird | Non_domestic | 1 | Wild |
| Great frigatebird | Bird | Non_domestic | 2 | Wild |
| Great hammerhead shark | Fish | Non_domestic | 2 | Wild |
| Great reed warbler | Bird | Non_domestic | 4 | Wild |
| Great roundleaf bat | Mammal | Non_domestic | 1 | Captive, Wild |
| Great scallop | Invertebrate | Non_domestic | 4 | Captive, Wild |
| Great sculpin | Fish | Non_domestic | 2 | Captive |
| Great snipe | Bird | Non_domestic | 1 | Wild |
| Greater amberjack | Fish | Non_domestic | 1 | Captive |
| Greater white-fronted goose | Bird | Non_domestic | 2 | Wild |
| Greek tortoise | Reptile | Non_domestic | 1 | Wild |
| Green sturgeon | Fish | Non_domestic | 2 | Captive, Wild |
| Green turtle | Reptile | Non_domestic | 19 | Captive, Wild |
| Greenland shark | Fish | Non_domestic | 2 | Wild |
| Greenland white-fronted goose | Bird | Non_domestic | 1 | Wild |
| Grey reef shark | Fish | Non_domestic | 3 | Wild |
| Grey seal | Mammal | Non_domestic | 5 | Captive, Wild |
| Grey-headed albatross | Bird | Non_domestic | 1 | Wild |
| Greylag goose | Bird | Non_domestic | 3 | Captive |
| Grivet | Mammal | Non_domestic | 1 | Captive |
| Grizzly bear | Mammal | Non_domestic | 2 | Captive, Wild |
| Guanaco | Mammal | Non_domestic | 1 | Captive |
| Guinea fowl | Bird | Non_domestic | 2 | Captive |
| Guinea pig | Mammal | Domestic | 4 | Captive |
| Gummy shark | Fish | Non_domestic | 1 | Wild |
| Hairy-nosed wombat | Mammal | Non_domestic | 3 | Captive, Wild |
| Hammerheaded bat | Mammal | Non_domestic | 1 | Wild |
| Harbour porpoise | Mammal | Non_domestic | 6 | Captive, Wild |
| Harbour seal | Mammal | Non_domestic | 7 | Captive, Wild |
| Harris's hawk | Bird | Non_domestic | 2 | Captive, Wild |
| Hawaiian monk seal | Mammal | Non_domestic | 2 | Captive, Wild |
| Hawksbill turtle | Reptile | Non_domestic | 4 | Captive, Wild |
| Hawksmoth | Invertebrate | Non_domestic | 1 | Captive |
| Herring gull | Bird | Non_domestic | 4 | Wild |
| Himalayan griffon vulture | Bird | Non_domestic | 5 | Captive, Wild |
| Hoary bat | Mammal | Non_domestic | 1 | Wild |
| Hog-nosed skunk | Mammal | Non_domestic | 1 | Captive |
| Hooded seal | Mammal | Non_domestic | 2 | Captive |
| Horn shark | Fish | Non_domestic | 2 | Wild |
| Horse | Mammal | Domestic | 55 | Captive |
| Humpback whale | Mammal | Non_domestic | 19 | Wild |
| Iberian lynx | Mammal | Non_domestic | 1 | Wild |
| Impala | Mammal | Non_domestic | 1 | Wild |
| Imperial cormorant | Bird | Non_domestic | 14 | Captive, Wild |
| Indo-Pacific bottlenose dolphin | Mammal | Non_domestic | 2 | Captive |
| Indo-Pacific sailfish | Fish | Non_domestic | 1 | Wild |
| Jaguar | Mammal | Non_domestic | 1 | Captive |
| Jaguarundi | Mammal | Non_domestic | 1 | Captive |
| Japanese amberjack | Fish | Non_domestic | 2 | Captive |
| Japanese flounder | Fish | Non_domestic | 3 | Captive, Wild |
| Japanese lates | Fish | Non_domestic | 2 | Captive, Wild |
| Japanese macaque | Mammal | Non_domestic | 2 | Captive |
| Japanese quail | Bird | Non_domestic | 2 | Captive |
| Japanese sea bass | Fish | Non_domestic | 1 | Captive |
| Javan gibbon | Mammal | Non_domestic | 1 | Captive |
| Javan slow loris | Mammal | Non_domestic | 1 | Wild |
| Jonah crab | Invertebrate | Non_domestic | 1 | Captive |
| Keel-billed toucan | Bird | Non_domestic | 1 | Wild |
| Kerguelen shag | Bird | Non_domestic | 2 | Wild |
| Khulan | Mammal | Non_domestic | 1 | Wild |
| Killer whale | Mammal | Non_domestic | 9 | Captive, Wild |
| King penguin | Bird | Non_domestic | 9 | Captive, Wild |
| Koala | Mammal | Non_domestic | 3 | Captive, Wild |
| Lake sturgeon | Fish | Non_domestic | 3 | Wild |
| Lake trout | Fish | Non_domestic | 2 | Captive, Wild |
| Lappet-faced vulture | Bird | Non_domestic | 1 | Wild |
| Larga seal | Mammal | Non_domestic | 1 | Captive |
| Large hairy armadillo | Mammal | Non_domestic | 2 | Captive |
| Largemouth bass | Fish | Non_domestic | 1 | Wild |
| Larger hairy armadillo | Mammal | Non_domestic | 1 | Captive |
| Largetooth sawfish | Fish | Non_domestic | 3 | Captive, Wild |
| Laysan albatross | Bird | Non_domestic | 1 | Wild |
| Leatherback turtle | Reptile | Non_domestic | 4 | Wild |
| Lemon shark | Fish | Non_domestic | 15 | Captive, Wild |
| Lesser black-backed gull | Bird | Non_domestic | 10 | Wild |
| Lesser kestrel | Bird | Non_domestic | 2 | Wild |
| Little bustard | Bird | Non_domestic | 1 | Wild |
| Little penguin | Bird | Non_domestic | 12 | Captive, Wild |
| Llama | Mammal | Non_domestic | 1 | Captive |
| Locust | Invertebrate | Non_domestic | 1 | Captive |
| Lodgepole chipmunk | Mammal | Non_domestic | 2 | Captive, Wild |
| Loggerhead turtle | Reptile | Non_domestic | 15 | Captive, Wild |
| Long finned squid | Invertebrate | Non_domestic | 1 | Captive |
| Long-finned pilot whale | Mammal | Non_domestic | 3 | Wild |
| Luderick | Fish | Non_domestic | 2 | Wild |
| Lusitanian toadfish | Fish | Non_domestic | 2 | Captive, Wild |
| Macaroni penguin | Bird | Non_domestic | 3 | Wild |
| Magellanic penguin | Bird | Non_domestic | 12 | Captive, Wild |
| Magnificent frigatebird | Bird | Non_domestic | 1 | Wild |
| Mahi-mahi | Fish | Non_domestic | 1 | Captive, Wild |
| Malayan colugo | Mammal | Non_domestic | 3 | Wild |
| Mallard | Bird | Non_domestic | 5 | Captive, Wild |
| Maned wolf | Mammal | Non_domestic | 1 | Captive |
| Manx shearwater | Bird | Non_domestic | 1 | Wild |
| Margay | Mammal | Non_domestic | 1 | Captive |
| Marmoset | Mammal | Non_domestic | 1 | Captive |
| Mediterranean mouflon | Mammal | Non_domestic | 2 | Captive, Wild |
| Mediterranean slipper lobster | Invertebrate | Non_domestic | 1 | Captive |
| Mediterranean tortoise | Reptile | Non_domestic | 1 | Captive |
| Meerkat | Mammal | Non_domestic | 3 | Wild |
| Milky stork | Bird | Non_domestic | 1 | Captive |
| Minke whale | Mammal | Non_domestic | 5 | Wild |
| Moon jellyfish | Invertebrate | Non_domestic | 1 | Captive |
| Moose | Mammal | Non_domestic | 4 | Wild |
| Mountain brushtail possum | Mammal | Non_domestic | 2 | Wild |
| Mouse | Mammal | Domestic | 8 | Captive |
| Mulloway | Fish | Non_domestic | 2 | Wild |
| Murray cod | Fish | Non_domestic | 2 | Wild |
| Muscovy duck | Bird | Non_domestic | 2 | Captive |
| Musk turtle | Reptile | Non_domestic | 1 | Wild |
| Muskellunge | Fish | Non_domestic | 1 | Wild |
| Muskoxen | Mammal | Non_domestic | 2 | Wild |
| Naked dragonfish | Fish | Non_domestic | 1 | Captive |
| Narwhal | Mammal | Non_domestic | 3 | Wild |
| Nazca boobie | Bird | Non_domestic | 1 | Wild |
| New Calidonian sea krait | Reptile | Non_domestic | 1 | Captive |
| New Zealand fur seal | Mammal | Non_domestic | 5 | Captive |
| North American beaver | Mammal | Non_domestic | 1 | Captive |
| North Atlantic right whale | Mammal | Non_domestic | 7 | Wild |
| Northern bald ibis | Bird | Non_domestic | 1 | Captive, Wild |
| Northern bottlenose whale | Mammal | Non_domestic | 2 | Wild |
| Northern elephant seal | Mammal | Non_domestic | 16 | Captive, Wild |
| Northern fur seal | Mammal | Non_domestic | 12 | Captive, Wild |
| Northern gannet | Bird | Non_domestic | 4 | Wild |
| Northern pike | Fish | Non_domestic | 4 | Captive |
| Northern tamandua | Mammal | Non_domestic | 3 | Wild |
| Nurse shark | Fish | Non_domestic | 2 | Captive, Wild |
| Ocean sunfish | Fish | Non_domestic | 3 | Wild |
| Oceanic whitetip shark | Fish | Non_domestic | 1 | Wild |
| Ocelot | Mammal | Non_domestic | 2 | Captive, Wild |
| Oilbird | Bird | Non_domestic | 1 | Wild |
| Olive baboon | Mammal | Non_domestic | 3 | Wild |
| Oncilla | Mammal | Non_domestic | 1 | Captive |
| Osprey | Bird | Non_domestic | 1 | Wild |
| Ostrich | Bird | Non_domestic | 2 | Captive |
| Pacific bluefin tuna | Fish | Non_domestic | 2 | Captive |
| Pacific cod | Fish | Non_domestic | 1 | Captive |
| Pacific halibut | Fish | Non_domestic | 2 | Captive, Wild |
| Pacific marten | Mammal | Non_domestic | 1 | Wild |
| Pacific sailfish | Fish | Non_domestic | 1 | Wild |
| Pacific sea nettle | Invertebrate | Non_domestic | 1 | Captive, Wild |
| Painted turtle | Reptile | Non_domestic | 1 | Wild |
| Pallid swift | Bird | Non_domestic | 1 | Wild |
| Pampas cat | Mammal | Non_domestic | 1 | Captive |
| Peahen | Bird | Domestic | 1 | Captive |
| Pekin duck | Bird | Domestic | 4 | Captive |
| Pelagic cormorant | Bird | Non_domestic | 1 | Wild |
| Permit | Fish | Non_domestic | 1 | Wild |
| Pig | Mammal | Domestic | 49 | Captive |
| Pigeon | Bird | Non_domestic | 11 | Captive, Wild |
| Pileated gibbon | Mammal | Non_domestic | 1 | Captive |
| Pine marten | Mammal | Non_domestic | 1 | Wild |
| Pink-footed goose | Bird | Non_domestic | 1 | Wild |
| Plains zebra | Mammal | Non_domestic | 1 | Wild |
| Platypus | Mammal | Non_domestic | 1 | Wild |
| Polar bear | Mammal | Non_domestic | 10 | Captive, Wild |
| Port Jackson shark | Fish | Non_domestic | 2 | Captive, Wild |
| Prickly shark | Fish | Non_domestic | 1 | Wild |
| Puma | Mammal | Non_domestic | 10 | Captive, Wild |
| Pygmy blue whale | Mammal | Non_domestic | 1 | Wild |
| Queen conch | Invertebrate | Non_domestic | 3 | Wild |
| Rabbit | Mammal | Domestic | 8 | Captive, Wild |
| Rainbow trout | Fish | Non_domestic | 7 | Captive |
| Rat | Mammal | Domestic | 19 | Captive |
| Rattlesnake | Reptile | Non_domestic | 1 | Wild |
| Razorbill | Bird | Non_domestic | 3 | Wild |
| Red deer | Mammal | Non_domestic | 2 | Captive, Wild |
| Red fox | Mammal | Non_domestic | 3 | Captive, Wild |
| Red sea bream | Fish | Non_domestic | 2 | Captive |
| Red snapper | Fish | Non_domestic | 3 | Captive, Wild |
| Red squirrel | Mammal | Non_domestic | 3 | Wild |
| Red stingray | Fish | Non_domestic | 1 | Wild |
| Red-backed shrike | Bird | Non_domestic | 2 | Wild |
| Red-footed boobie | Bird | Non_domestic | 2 | Wild |
| Redfronted lemur | Mammal | Non_domestic | 1 | Wild |
| Red-legged kittiwake | Bird | Non_domestic | 1 | Wild |
| Red-ruffed lemur | Mammal | Non_domestic | 1 | Captive |
| Red-spotted grouper | Fish | Non_domestic | 1 | Captive |
| Red-tailed tropicbird | Bird | Non_domestic | 1 | Wild |
| Rhesus macaque | Mammal | Non_domestic | 14 | Captive |
| Rhinoceros auklet | Bird | Non_domestic | 1 | Wild |
| Ring ouzel | Bird | Non_domestic | 1 | Wild |
| Risso's dolphin | Mammal | Non_domestic | 7 | Captive, Wild |
| Rockhopper penguin | Bird | Non_domestic | 2 | Captive |
| Rocky Mountain elk | Mammal | Non_domestic | 4 | Captive |
| Roe deer | Mammal | Non_domestic | 9 | Captive, Wild |
| Rothschild's giraffe | Mammal | Non_domestic | 1 | Captive |
| Rough-legged buzzard | Bird | Non_domestic | 1 | Wild |
| Saithe | Fish | Non_domestic | 2 | Captive |
| Sakhalin taimen | Fish | Non_domestic | 1 | Wild |
| Saltwater crocodile | Reptile | Non_domestic | 1 | Wild |
| Sand tiger | Fish | Non_domestic | 1 | Captive, Wild |
| Sand whiting | Fish | Non_domestic | 2 | Wild |
| Sandbar shark | Fish | Non_domestic | 6 | Captive, Wild |
| Satyr tragopan | Bird | Non_domestic | 1 | Wild |
| Scalloped hammerhead shark | Fish | Non_domestic | 3 | Wild |
| Scimitar-horned oryx | Mammal | Non_domestic | 1 | Captive |
| Scopoli's shearwater | Bird | Non_domestic | 2 | Wild |
| Sei whale | Mammal | Non_domestic | 2 | Wild |
| Sheep | Mammal | Domestic | 58 | Captive |
| Shore crab | Invertebrate | Non_domestic | 1 | Captive |
| Short-beaked echidna | Mammal | Non_domestic | 2 | Captive, Wild |
| Short-finned pilot whale | Mammal | Non_domestic | 4 | Wild |
| Shorthorn Sculpin | Fish | Non_domestic | 1 | Wild |
| Shortnose sturgeon | Fish | Non_domestic | 2 | Captive, Wild |
| Short-tailed shearwater | Bird | Non_domestic | 1 | Wild |
| Sicklefin lemon shark | Fish | Non_domestic | 1 | Wild |
| Signal crayfish | Invertebrate | Non_domestic | 2 | Captive |
| Silver redhorse | Fish | Non_domestic | 1 | Wild |
| Smallmouth bass | Fish | Non_domestic | 4 | Captive, Wild |
| Smalltooth sawfish | Fish | Non_domestic | 2 | Wild |
| Snapping turtle | Reptile | Non_domestic | 1 | Wild |
| Snowshoe hare | Mammal | Non_domestic | 3 | Wild |
| Sockeye salmon | Fish | Non_domestic | 7 | Captive, Wild |
| Sooty albatross | Bird | Non_domestic | 1 | Wild |
| South American sea lion | Mammal | Non_domestic | 1 | Wild |
| South Georgia shag | Bird | Non_domestic | 1 | Wild |
| Southern elephant seal | Mammal | Non_domestic | 29 | Wild |
| Southern giraffe | Mammal | Non_domestic | 1 | Wild |
| Southern right whale | Mammal | Non_domestic | 1 | Wild |
| Southern stingray | Fish | Non_domestic | 1 | Wild |
| Southern woolly lemur | Mammal | Non_domestic | 1 | Wild |
| Sperm whale | Mammal | Non_domestic | 12 | Wild |
| Spider monkey | Mammal | Non_domestic | 2 | Captive |
| Spine-bellied sea snake | Reptile | Non_domestic | 2 | Captive, Wild |
| Spinner shark | Fish | Non_domestic | 1 | Wild |
| Spotted notothen | Fish | Non_domestic | 1 | Captive |
| Squirrel monkey | Mammal | Non_domestic | 1 | Captive |
| Steelhead trout | Fish | Non_domestic | 1 | Wild |
| Steller sea lion | Mammal | Non_domestic | 11 | Captive, Wild |
| Straw-coloured fruit bat | Mammal | Non_domestic | 3 | Wild |
| Streaked shearwater | Bird | Non_domestic | 5 | Wild |
| Striated caracaras | Bird | Non_domestic | 1 | Wild |
| Striped bass | Fish | Non_domestic | 1 | Captive |
| Striped rockcod | Fish | Non_domestic | 1 | Captive |
| Subantarctic fur seal | Mammal | Non_domestic | 3 | Captive |
| Sunset rockfish | Fish | Non_domestic | 1 | Wild |
| Svalbard reindeer | Mammal | Non_domestic | 3 | Wild |
| Swainson's thrush | Bird | Non_domestic | 1 | Captive |
| Swell shark | Fish | Non_domestic | 1 | Wild |
| Tarwhine | Fish | Non_domestic | 1 | Wild |
| Tawny eagle | Bird | Non_domestic | 1 | Captive |
| Tawny pipit | Bird | Non_domestic | 1 | Wild |
| Thick-billed murre | Bird | Non_domestic | 5 | Wild |
| Three-toed sloth | Mammal | Non_domestic | 2 | Captive, Wild |
| Tiger flathead | Fish | Non_domestic | 1 | Wild |
| Tiger shark | Fish | Non_domestic | 7 | Wild |
| Tigerfish | Fish | Non_domestic | 1 | Wild |
| Tree shrew | Mammal | Non_domestic | 1 | Captive |
| Trochus | Invertebrate | Non_domestic | 1 | Wild |
| Turkey | Bird | Domestic | 4 | Captive |
| Veiled chameleon | Reptile | Non_domestic | 1 | Captive |
| Veined squid | Invertebrate | Non_domestic | 1 | Captive |
| Verreaux's sifaka | Mammal | Non_domestic | 2 | Captive, Wild |
| Vervet monkey | Mammal | Non_domestic | 1 | Wild |
| Wandering albatross | Bird | Non_domestic | 1 | Wild |
| Waterbuck | Mammal | Non_domestic | 2 | Wild |
| Waved albatross | Bird | Non_domestic | 1 | Wild |
| Weddell seal | Mammal | Non_domestic | 14 | Wild |
| West Indian manatee | Mammal | Non_domestic | 1 | Captive |
| Western sandpiper | Bird | Non_domestic | 3 | Captive |
| Western tarsier | Mammal | Non_domestic | 1 | Wild |
| Whale shark | Fish | Non_domestic | 10 | Wild |
| White rhinoceros | Mammal | Non_domestic | 2 | Captive |
| White shark | Fish | Non_domestic | 9 | Wild |
| White stork | Bird | Non_domestic | 8 | Wild |
| White sturgeon | Fish | Non_domestic | 1 | Wild |
| White-backed vulture | Bird | Non_domestic | 1 | Wild |
| White-chinned petrel | Bird | Non_domestic | 1 | Wild |
| White-fronted goose | Bird | Non_domestic | 3 | Captive, Wild |
| White-spotted conger | Fish | Non_domestic | 1 | Captive |
| White-streaked grouper | Fish | Non_domestic | 2 | Captive |
| White-tailed deer | Mammal | Non_domestic | 1 | Captive, Wild |
| Whitetip reef shark | Fish | Non_domestic | 2 | Captive, Wild |
| Wild boar | Mammal | Non_domestic | 5 | Captive, Wild |
| Wild turkey | Bird | Non_domestic | 1 | Wild |
| Wolf | Mammal | Non_domestic | 5 | Captive, Wild |
| Wolverine | Mammal | Non_domestic | 3 | Captive, Wild |
| Wood sandpiper | Bird | Non_domestic | 1 | Captive, Wild |
| Yangtze finless porpoise | Mammal | Non_domestic | 4 | Wild |
| Yellowfin bream | Fish | Non_domestic | 3 | Wild |
| Yellowtail kingfish | Fish | Non_domestic | 3 | Captive, Wild |
| Zebra finch | Bird | Non_domestic | 3 | Captive |
| Zebu | Mammal | Non_domestic | 1 | Captive |

**Supplementary Table 5:** Table of median sample size by taxon and research question. Text in brackets represents total number of studies

| Research topic | Captive | | | | |  | Wild | | | | |
| --- | --- | --- | --- | --- | --- | --- | --- | --- | --- | --- | --- |
|  | Mammal | Bird | Fish | Reptile | Invertebrate |  | Mammal | Bird | Fish | Reptile | Invertebrate |
| Behavioural studies | 15  (418) | 12  (43) | 10  (49) | 8  (8) | 13  (13) |  | 11  (253) | 17  (104) | 9  (73) | 6  (21) | 17  (10) |
| Technology & Methods | 10  (288) | 10  (34) | 7  (29) | 4  (11) | 11  (10) |  | 7.5  (93) | 12  (46) | 7  (28) | 2  (18) | 14.5  (2) |
| Environmental & Ecological studies | 20  (44) | 18  (11) | 12  (20) | 22  (3) | 37  (2) |  | 12  (112) | 19  (70) | 10  (46) | 5  (10) | 9  (5) |
| Movement & Biomechanics | 9  (95) | 21  (27) | 7.5  (17) | 4  (5) | 8  (7) |  | 9  (106) | 14  (81) | 6  (34) | 4  (10) | 7.5  (2) |
| Energy expenditure & Physiology | 8.5  (83) | 8  (18) | 15.5  (35) | 8  (7) | 11  (7) |  | 9.5  (72) | 17  (36) | 9  (38) | 2.5  (8) | 18.5  (2) |
| Welfare, Health & Husbandry | 28  (211) | 48  (19) | 16.5  (16) | 33  (1) | NA |  | 23.5  (14) | 27  (10) | 27  (17) | 64  (1) | 21  (3) |
| Human impact | 11  (6) | 21  (5) | 17  (5) | NA | 24  (6) |  | 13  (37) | 17.5  (8) | 27  (8) | 16  (3) | 20  (1) |
| Neuroscience & Medicine | 8.5  (58) | 4  (5) | 1  (1) | 6  (1) | 10.5  (2) |  | 4  (2) | NA | NA | NA | NA |
